# PrrT/A, a Pseudomonas aeruginosa Bacterial Encoded Toxin-Antitoxin System Involved in Prophage Regulation and Biofilm Formation

**DOI:** 10.1128/spectrum.01182-22

**Published:** 2022-05-16

**Authors:** Esther Shmidov, Ilana Lebenthal-Loinger, Shira Roth, Sarit Karako-Lampert, Itzhak Zander, Sivan Shoshani, Amos Danielli, Ehud Banin

**Affiliations:** a The Mina and Everard Goodman Faculty of Life Sciences, Bar-Ilan University, Ramat-Gan, Israel; b The Institute of Nanotechnology and Advanced Materials, Bar-Ilan University, Ramat Gan, Israel; c Scientific Equipment Center, The Mina & Everard Goodman Faculty of Life Sciences Bar-Ilan University, Ramat Gan, Israel; d Faculty of Engineering, Bar-Ilan University, Ramat Gan, Israel; University Roma Tre

**Keywords:** *Pseudomonas aeruginosa*, toxin-antitoxin, biofilm, prophages, bacteriophages, biofilms

## Abstract

Toxin-antitoxin (TA) systems are genetic modules that consist of a stable protein-toxin and an unstable antitoxin that neutralizes the toxic effect. In type II TA systems, the antitoxin is a protein that inhibits the toxin by direct binding. Type II TA systems, whose roles and functions are under intensive study, are highly distributed among bacterial chromosomes. Here, we identified and characterized a novel type II TA system PrrT/A encoded in the chromosome of the clinical isolate 39016 of the opportunistic pathogen Pseudomonas aeruginosa. We have shown that the PrrT/A system exhibits classical type II TA characteristics and novel regulatory properties. Following deletion of the *prrA* antitoxin, we discovered that the system is involved in a range of processes including (i) biofilm and motility, (ii) reduced prophage induction and bacteriophage production, and (iii) increased fitness for aminoglycosides. Taken together, these results highlight the importance of this toxin-antitoxin system to key physiological traits in P. aeruginosa.

**IMPORTANCE** The functions attributed to bacterial TA systems are controversial and remain largely unknown. Our study suggests new insights into the potential functions of bacterial TA systems. We reveal that a chromosome-encoded TA system can regulate biofilm and motility, antibiotic resistance, prophage gene expression, and phage production. The latter presents a thus far unreported function of bacterial TA systems. In addition, with the emergence of antimicrobial-resistant bacteria, especially with the rising of P. aeruginosa resistant strains, the investigation of TA systems is critical as it may account for potential new targets against the resistant strains.

## INTRODUCTION

Toxin-antitoxin (TA) systems are genetic modules that were initially identified as plasmid maintenance systems (postsegregational killing) (1, 2). Over the last few decades, thousands of TA loci were identified on plasmids, phages, and bacterial and Archael and chromosomes, harboring different functions (3, 4). The TA locus encodes for a toxin and a relatively unstable cognate antitoxin that neutralizes the toxic effect during normal bacterial growth (5). While the toxin gene typically encodes for a protein, the antitoxin gene product can be either an RNA or a low-molecular-weight protein, depending on the TA class (4). Type II TA system genes are commonly expressed under one bicistronic operon by a tightly autoregulated promoter (6), and the antitoxin is a protein that neutralizes the toxin by direct binding (7). The antitoxin usually negatively regulates the operon transcription by direct DNA binding, with relatively low affinity that can be enhanced by antitoxin-toxin complexes ratio (8). The activation of type II TA systems also undergoes posttranslational regulation, dependent on the antitoxin instability and degradation mediated by bacterial proteases (9). The proteolysis of the antitoxin promotes under several stress conditions, leading to the activation of the TA system (10).

The Type II TA class is considered the most abundant system in bacterial genomes and was identified across diverse bacterial species (6). As the original plasmid-stabilization function is not relevant for the diverse TA systems found in bacterial chromosomes, TA systems are hypothesized to be involved in other biological processes, for example, stabilizing chromosomal mobile elements (11, 12), bacteriophage inhibition by abortive infection (13), involvement in bacterial stress response, persisters formation (14, 15), and biofilm formation (16). The diverse functions of type II TA systems and their role in bacterial responses are extensively studied yet remain largely unknown (17).

Pseudomonas aeruginosa is a Gram-negative, rod-shaped bacterium with a single flagellum. It is an opportunistic pathogen of plants, nematodes, insects, animals, and humans (18). It can cause a wide range of acute and chronic infections, enhanced by the bacterium's low susceptibility to various antimicrobial substances, making most of the infections difficult to treat and life-threatening (19). Several type II TA systems were previously identified in the genome of P. aeruginosa*;* three were shown bioinformatically to be highly conserved between P. aeruginosa isolates; *parE-parD* (20), *relE-relB* (21), and *higB-higA* (22).

The ParE class of toxins acts through direct binding and inhibition of DNA gyrase, resulting in accumulation of DNA breaks, activation of SOS response, and bacterial death (17, 20). HigB toxin acts as an RNase, which rapidly degrades mRNAs and influences bacterial virulence by enhancing the type III secretion system (23), reducing pyochelin and pyocyanin production, biofilm formation, and swarming motility (22). HigA functions as an antitoxin that neutralizes the RNase activity of HigB (22). The *higA* gene has an independent promoter apart from the joint *higB/A* promoter, resulting in higher *higA* transcripts in the late stationary phase (24). Moreover, besides the auto-repression properties of HigA, it also binds and represses the *mvfR* promoter, a central virulence transcription regulator (24).

In the current study, we identified and characterized a novel type II TA system PrrT/A (named for Prophage Regulator Toxin/Antitoxin) encoded in the chromosome of the clinical isolate 39016 of P. aeruginosa. The toxin PrrT, carrying a ParE-like domain, is inhibited by PrrA, an antitoxin with a predicted HigA-like domain. We have found that the PrrT/A system affects bacterial growth, biofilm formation, swarming motility, prophage induction, bacteriophage production, and aminoglycosides fitness.

## RESULTS

### PrrT/A exhibits classical type II TA characteristics.

PA39016_100004 (*prrA*) gene product is a DNA binding protein with a predicted HigA-like domain. The sequence of the 39016 strain is poorly annotated, and many open reading frames (ORFs) are not properly detected in the sequence. To better understand the functionality of the *prrA* gene, the genomic region of *prrA* was scanned to detect unannotated genes that might influence the *prrA* gene function. The screening identified one such gene, 366 bp ORF adjacent to *prrA*, herein termed *prrT*. The protein sequence analysis revealed that the *prrT* product has a ParE-like domain, suggesting that PrrT and PrrA act together as a type II TA system.

**FIG 1:**
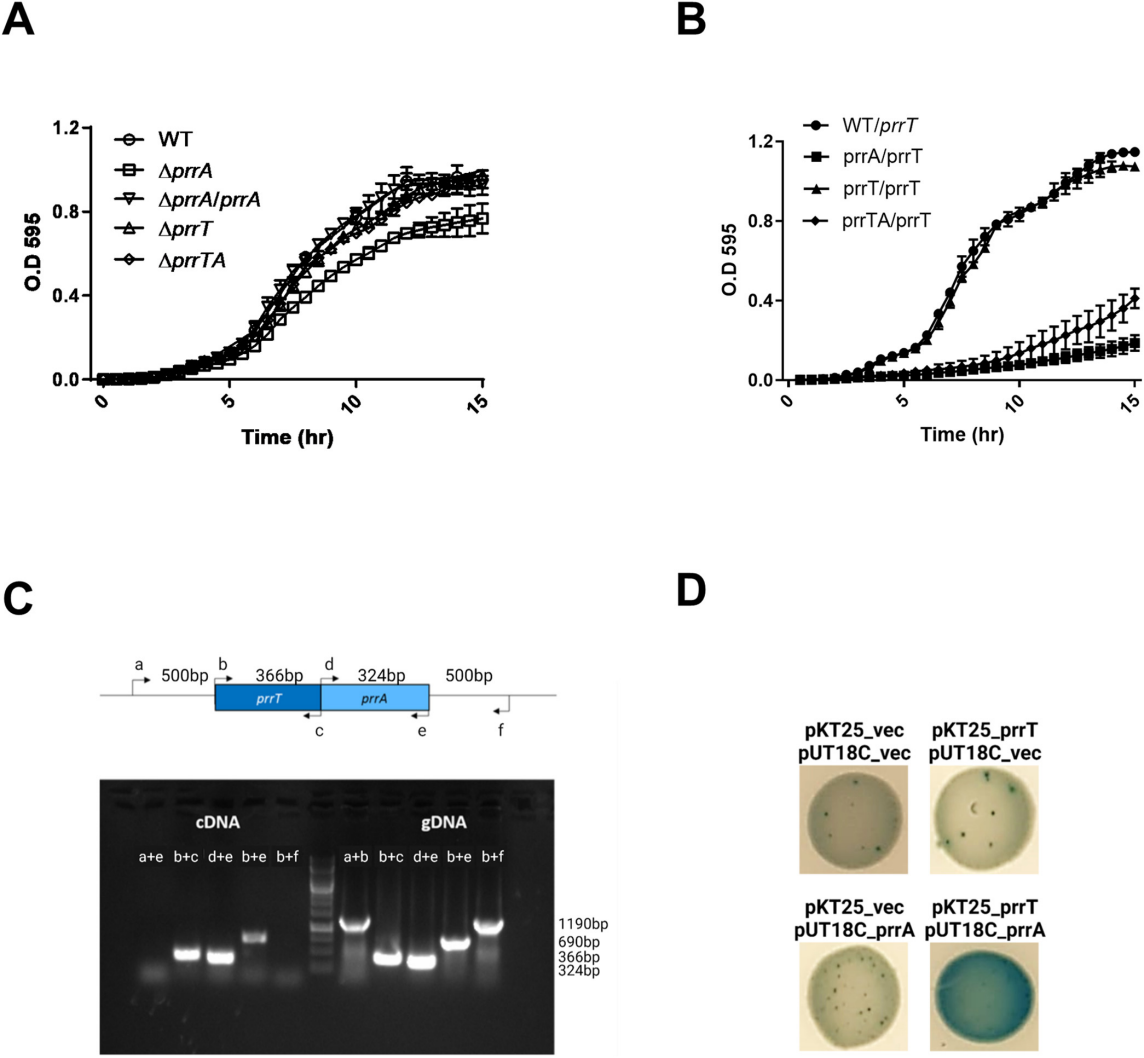
The *prrT/prrA* gene pair act as a type II TA system. (A) Deletion of *prrA* in 39016 strain revealed a decrease in the growth rate; the growth curve of the mutants in comparison to the WT, and complementation by arabinose induced *prrA* expression in the mutant. (B) *prrT* expression is toxic only for the Δ*prrA* strain; the growth curve of *prrT* induced OE in the WT and mutant strains, and the inducer was added immediately after the dilution. (C) *prrT/prrA* gene pair expressed as a polycistron; the following sets of primers were used for the operon verification: (a) 500 bp upstream to *prrT*, (b) prrT_F, (c) prrT_R, (d) prrA_F, (e) prrA_R, and (f) 500 bp downstream to *prrA*. The cDNA results represent the transcripts, while gDNA results represent the bacterial genome as a positive control. (D) PrrT/PrrA form a protein complex together; BACTH assay on indicative LB plates containing IPTG and x-gal. The above (A) and (B) graphs are the averages of three independent experiments consisting of five replicates each. Error bars represent the standard deviations.

To investigate whether the *prrT/A* gene pair indeed encodes for a TA system, deletion mutants of the *prrA* (Δ*prrA*), *prrT* (Δ*prrT*), and both genes (Δ*prrTA*) were created. The growth curve of the different mutants showed that the deletion of the *prrA* gene significantly decreased the bacterial growth rate. The double mutant strain did not show any significant change in its growth, indicating that the growth inhibition depends on PrrT toxin activity. Complementation by a genomic expressed copy of *prrA* (Δ*prrA*/*prrA*) restored the bacterial growth phenotype with no significant difference to the wild-type (WT) strain (Fig. 1A and Fig. S1A).

To examine the toxicity of PrrT, we inserted into an *attTn7* site of the WT and mutant strains an intact copy of the *prrT* gene under arabinose inducible promoter. PrrT induction did not affect the WT strain, while in both the Δ*prrA* and Δ*prrTA* strains, the toxin induction resulted in significant growth inhibition (Fig. 1B and Fig. S1B). The recovery rate of the Δ*prrTA* strain was somewhat higher than Δ*prrA*, probably due to an unneutralized native toxin present in the Δ*prrA* strain.

Type II TA systems genes are commonly expressed as a bicistronic mRNA (25). To verify whether this is also the case for the *prrTA*, systemRNA was extracted from the WT 39016, reverse transcribed to cDNA, and was further amplified by PCR with specific primers for the pair of genes compared to genomic DNA (gDNA). The results showed that *prrT* and *prrA* are expressed by polycistronic mRNA, reinforcing our hypothesis that they act as a type II TA system (Fig. 1C).

As the type II TA system neutralization mechanism is mediated by specific interaction between the toxin and the antitoxin, we also examined the PrrT-PrrA binding. Both classical Co-IP and BACTH assays strongly showed that the proteins directly interact and form a complex (Fig. 1D, Fig. S2).

### PrrA protein represses *prrT/A* operon expression.

**FIG 2:**
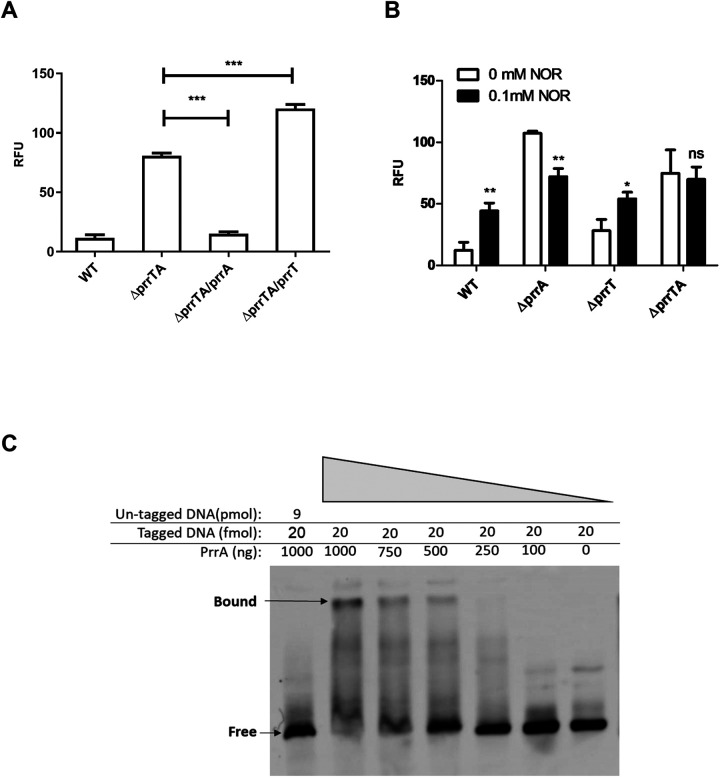
The *prrT/A* promoter activity is affected by PrrA, PrrT, and NOR stressor. (A) PrrA represses the promoter while PrrT expression elevates the activity; fluorescence measurement of the WT and the ΔprrTA strain with single complementation, and PrrA and PrrT were induced in the *ΔprrTA* strain at time zero with 10 mM arabinose. (B) NOR treatment elevated the promoter activity exclusively in strains with an intact *prrA*; fluorescence measurement of the WT and mutant strains with or without 0.1 mM NOR treatment at time zero. (C) PrrA directly binds to the prrT/A promoter; competition sample with biotinylated DNA and an unlabeled competitor DNA (lane 1), biotinylated DNA with the addition of decreasing amount of the PrrA protein (lanes 2–7). For the A and B graphs, m-Cherry fluorescence measurements were taken in the early stationary phase. The above A and B graphs are the average of three independent experiments consisting of five replicates each. Error bars represent the standard deviation. According to *t* test: *, *P* < 0.05; **, *P* < 0.01.

The PrrA antitoxin is a predicted transcriptional regulator as it contains a DNA binding domain. To characterize its regulatory properties, self-regulation was evaluated by examining the *prrT/A* promoter activity in different mutant strains. For that, a transcription fusion was constructed. Briefly, 300 bp upstream to *prrT* were amplified, fused to *mCherry* reporter, and inserted into the *attCtx* site in the WT and mutant strains; the promoter activity was compared in the early stationary phase, 14 h postdilution. As expected, in the WT strain, the activity of the promoter was significantly decreased compared to the Δ*prrAT* operon mutant, indicating negative regulation of the proteins (Fig. 2A). To better characterize the contribution of each protein to the autorepression, single complementation strains, created by inserting an inducible copy of *prrT* or *prrA* into the *attTn7* site of Δ*prrTA*/mCherry strain, were utilized. The fluorescent measurements of these strains clearly showed that PrrA expression caused significant promoter repression while the toxin induction resulted in promoter activity elevation (Fig. 2A). These results strongly suggest that the PrrA protein is sufficient for the operon autorepression, while in the absence of the antitoxin, PrrT can stimulate auto-expression.

Type II TA systems are activated under different stress conditions, presumably due to the antitoxin cleavage (26). To confirm that the toxin effect on the promoter is not due to growth inhibition and/or stress conditions, we tested the Δ*prrT* strain exposed to different stressors. Out of all the tested stress conditions, only treatment with subinhibitory Norfloxacin (NOR) concentrations resulted in a significant increase of promoter activity in the WT strain (Fig. S3). We hypothesized that the increase in promoter activity depends on PrrA cleavage. Examination of the effect of NOR treatment on the different strains verified the antitoxin-dependent regulation. The treatment elevated the promoter activity only in strains harboring the *prrA* gene, while the treatment on the Δ*prrTA* strain showed no effect. The Δ*prrA* showed a decrease in fluorescence, perhaps due to the growth inhibition (Fig. 2B). The results reinforce the indication for the stress-independent positive regulation of PrrT, especially when considering that its effect was observed in the Δ*prrTA* strain.

Next, to confirm that the antitoxin represses the promoter by direct binding, electrophoretic mobility shift assay (EMSA) was carried out using a purified PrrA protein with 300 bp promoter region upstream to *prrT* start codon (*prrT/A* promoter). The results showed a clear shift in the sample containing both the tagged promoter and PrrA, indicating positive interaction between the antitoxin and the *prrT/A* promoter. The observed shift disappears with the addition of the competitor (an untagged DNA X120 excess) similar to the negative control, which does not contain the protein (Fig. 2C).

### PrrT/A is involved in biofilm and motility regulation.

**FIG 3:**
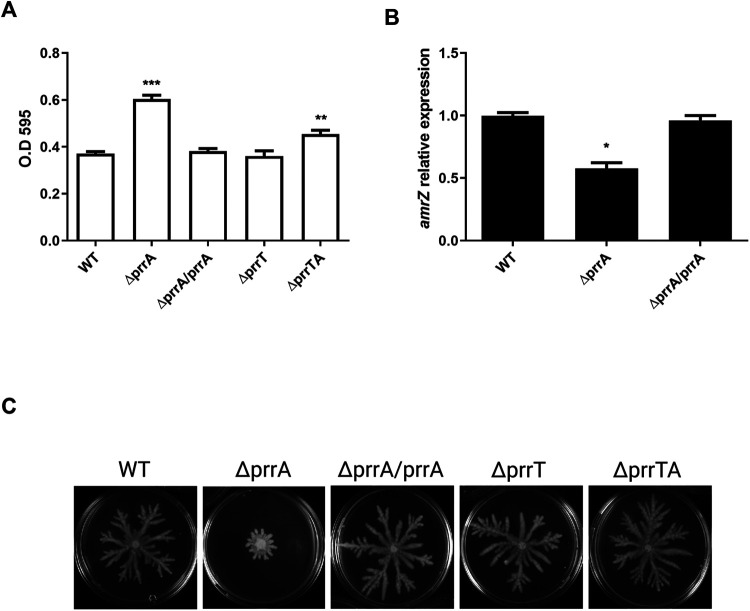
*prrA* deletion influenced swarming motility and biofilm formation. (A) *prrA* deletion significantly increased biofilm formation; CV stained 24 h biofilm of the strains. (B) The deletion of *prrA* significantly impacts the *amrZ* gene; RT- PCR analysis comparing the expression levels of *amrZ* the IN *prrA* mutant strain compared to the WT and complementation strains. (C) *prrA* deletion reduced swarming motility; the WT, mutants, and complementation strains were grown for 48 h. The A graph is the average of three independent experiments with five replicates each. The B graph is the average of three independent experiments with three replicates each. The C pictures represent three independent experiments conducted with three replicates each. Error bar represents standard error. **, *P* < 0.01 with WT strain as a reference, according to *t* test.

The impact of HigA antitoxin on biofilm regulation was previously published (22). To investigate whether PrrA influences biofilm formation and bacterial motility, the biofilm formation of the different mutant strains was quantified by crystal violet (CV) staining. Biofilm formation was significantly increased in the antitoxin mutant after 24 h, while the toxin mutant did not exhibit any difference compared to the WT strain (Fig. 3A). The double mutant strain showed nearly complete complementation with a significant decrease in comparison with the Δ*prrA* strain. Complementation by a genomic expressed copy of *prrA* completely restored the biofilm phenotype. Since some growth differences were detected after 24 h, we have also examined the biofilm formation with an extended incubation period (48 h). No planktonic growth differences were seen after 48 h, while the biofilm results were consistent with the shorter incubation time point (Fig. S4).

To understand how the antitoxin deletion influences biofilm formation, we performed a transcriptomic analysis of the WT and the Δ*prrA* strains. Focus was given to biofilm-related genes, influencing the bacterial cyclic di-GMP (c-di-GMP) levels. In P. aeruginosa, E. coli, and other species, the c-di-GMP second messenger regulates the switch between planktonic and biofilm growth (27). Two protein families mainly regulate the bacterial c-di-GMP levels: the diguanylate cyclase genes (DGCs) that promote c-di-GMP synthesis (28), and the phosphodiesterase genes (PDEs) that are involved in the turnover of cyclic-di-GMP (29). The analysis revealed a significant decrease in the transcript levels of the *amrZ* gene, a master regulator of several PDEs and DGCs genes (30). The results were validated by RT-PCR (Fig. 3B). Several downstream DGCs and PDEs were also affected, and their transcripts levels were altered correspondingly with the observed phenotype (Fig. S5). The results indicate that the observed increase in biofilm formation in the antitoxin mutant may result from elevated bacterial c-di-GMP levels.

Since AmrZ protein is a transcriptional factor that regulates motility and alginate synthesis (31), we further assessed the PrrA involvement in bacterial motility. The swarming of the different strains was examined (Fig. 3C). Consistently with the biofilm and the transcriptomic results, the Δ*prrA* strain exhibited significantly less swarming than the other strains. Unlike the biofilm phenotype, the double mutant strain showed complete restoration of the phenotype. Complementation by a genomic expressed *prrA* copy repaired the swarming phenotype.

We also examined the possible involvement of the PrrT/A system in bacterial persistence. The persistence assay showed that the WT and the *prrT/A* operon mutant showed similar persistence and the same biphasic death curve, indicating that the system is not involved in persistence formation (Fig. S6).

### PrrT/A is involved in prophage regulation.

The *prrA* gene was first identified by PHAST (32) within a prophage region in 39016. However, after the prophage *att* sites were identified (data not shown), it appeared that the gene is not a part of the prophage and is actually located 3,504 bp upstream. PrrA protein contains a CRO/CI-type DNA binding domain, so although proven to be a bacterial gene, we hypothesized that *prrA* might also be involved in prophage regulation.

**FIG 4:**
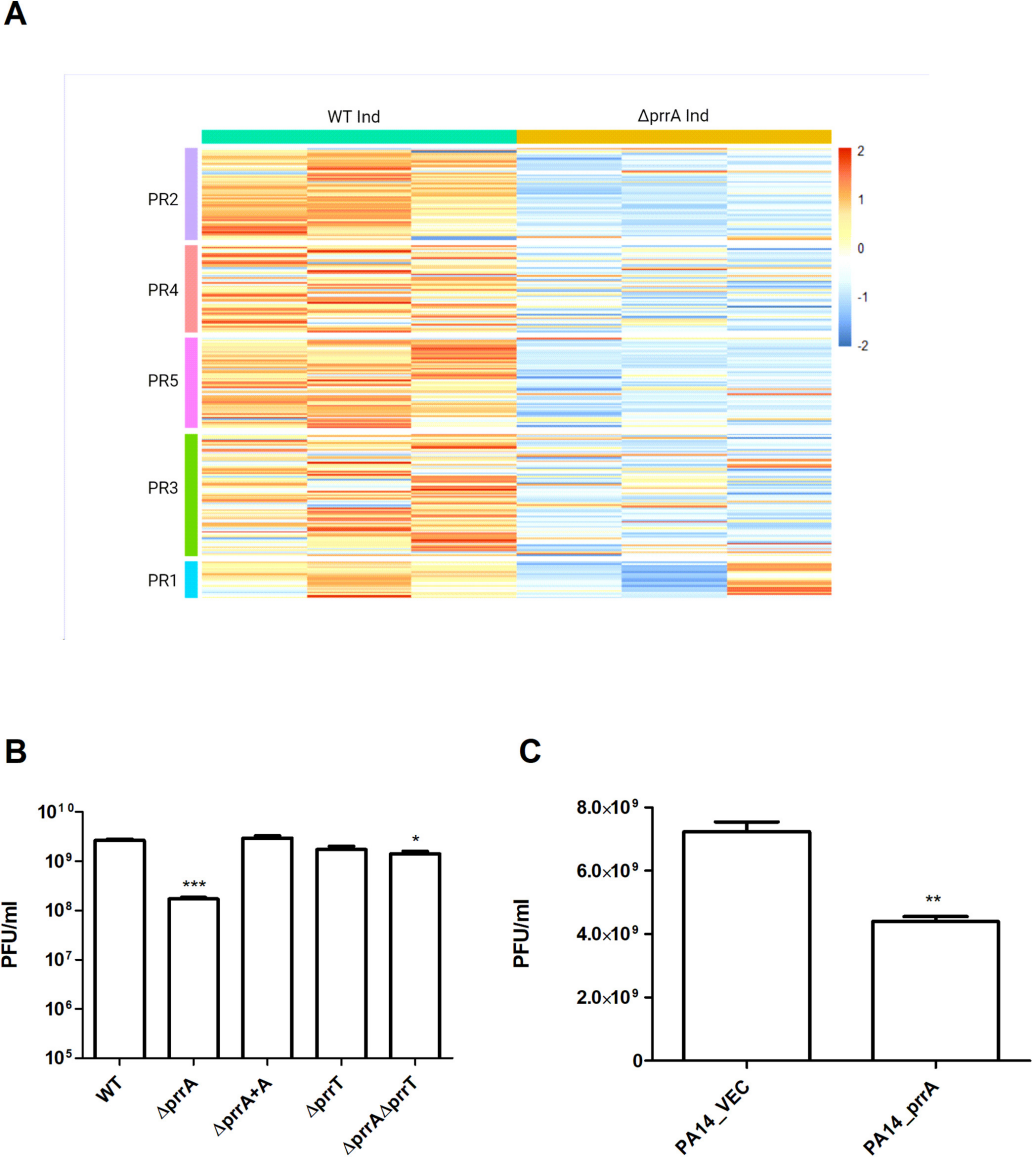
The *prrT*/*prrA* system is involved in prophage regulation and impacts phage production. (A) The *prrA* deletion resulted in a significant downregulation for most prophage encoded genes; heat-map was constructed with the RNA-seq results of the WT and Δ*prrA* strain, 1 h post prophage induction. The following prophage region (coordinates predicted by PHASTER [60]) genes were analyzed; PR1, PR2, PR3, PR4, and PR5. (B) The deletion of *prrA* resulted in decreased PR5 phage production; PA14 strain was used as a host, and phages were induced and extracted from WT, mutants, and the complementation strain. (C) *prrA* gene confers partial defense against phage infection; phages extracted from 39016 were used to infect the strains PA14/pUCP18 (PA14_VEC) and PAO1/pUCP18 *prrA* (PA14_*prrA*). The plaque-forming units presented in the above B and C graphs are the average of three independent experiments consisting of three replicates each. Error bars represent the standard deviations. *, *P* < 0.05; **, *P* < 0.01; ***, *P* < 0.001 when the WT and VEC is the reference strain, according to *t* test.

To examine this, we performed an RNA-seq analysis of the antitoxin mutant and the WT strains after induction for prophage excision (1 h postinduction) focusing on the genes encoded at the prophage regions of 39016, herein named PR1-5. The transcriptomic analysis revealed that the absolute majority of the “prophages” genes are significantly downregulated in the induced Δ*prrA* strain compared to the induced WT strain (Fig. 4A).

To further investigate whether the *prrA* deletion influenced phage production and infectivity, we quantified the phages produced by the mutants by performing a plaque assay with PA14 strain as a host. The results revealed that the Δ*prrA* strain produced significantly fewer infective phages than the WT strain (Fig. 4B). The Δ*prrT* had an equal number of phages as the WT, and the Δ*prrTA* strain produced slightly less than the WT strain yet significantly more than the Δ*prrA* strain.

The CRO/CI DNA binding domain of PrrA might indicate its involvement in lysogenic conversion or serve as an immunity element upon infection. To test this, *prrA* was overexpressed in the PA14 strain, and the susceptibility was examined and compared to PA14 carrying an empty vector. The overexpression (OE) of *prrA* resulted in a significant susceptibility reduction for phages extracted from the 39016 strain, indicating that the PrrA protein provides partial immunity for the strain (Fig. 4C).

### PrrT/A system affects bacterial fitness for subinhibitory concentrations of aminoglycosides.

**FIG 5:**
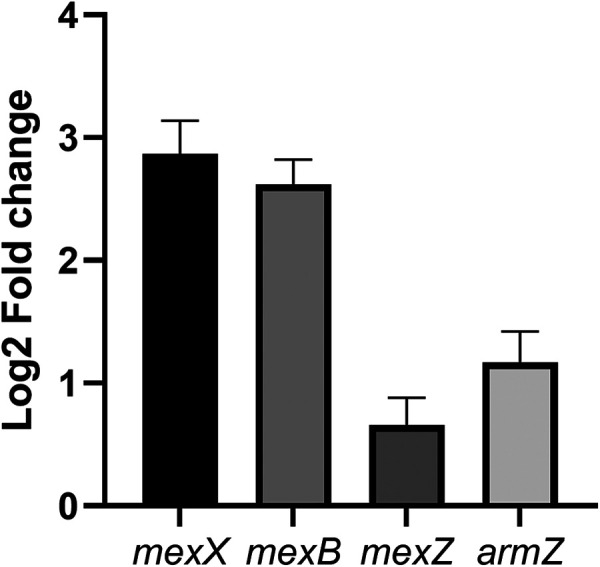
The MexXY system is upregulated in the *prrA* mutant. Log_2_ fold change of the mutant strain relative to the WT strain. Values were calculated with the transcriptomics levels. Error bars represent standard error.

**FIG 6:**
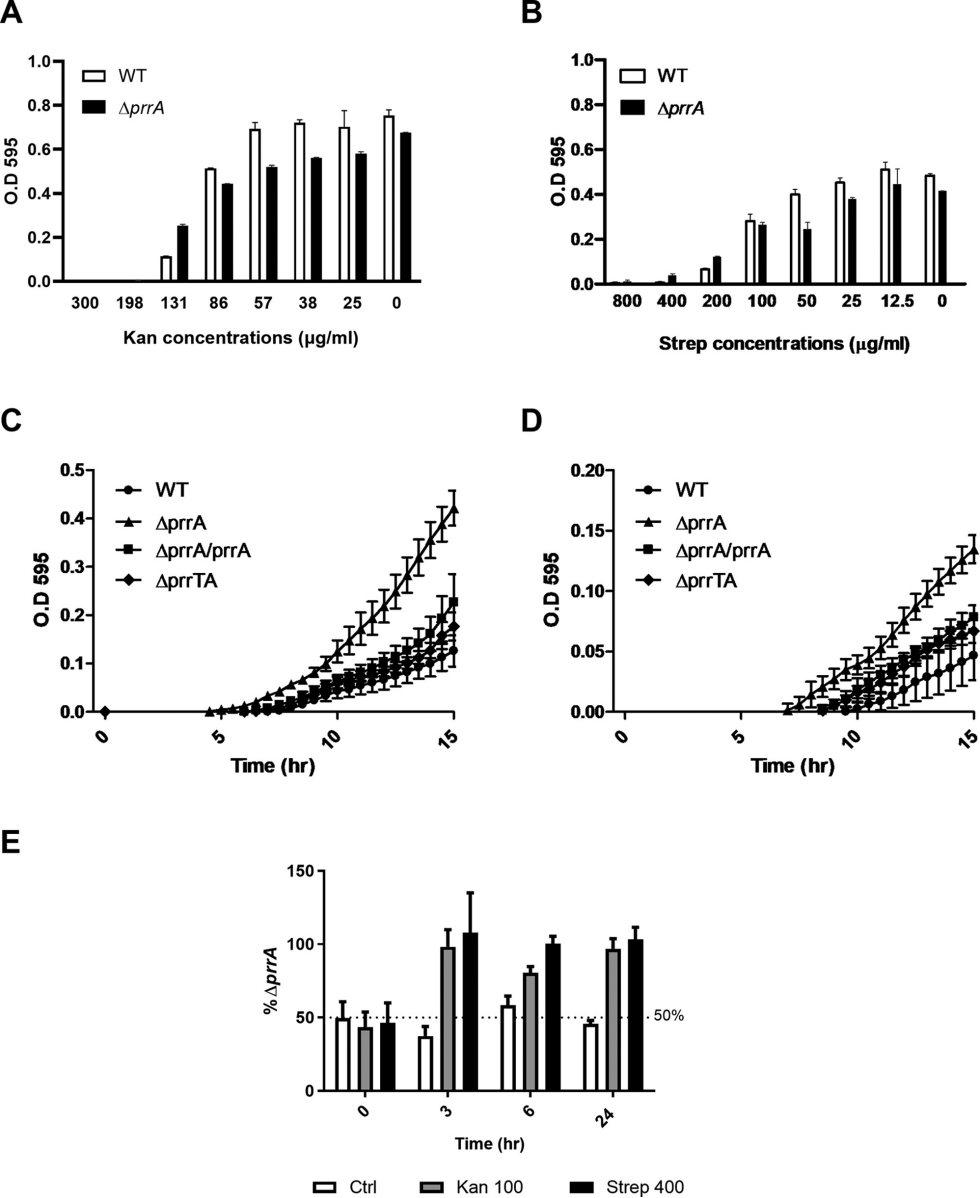
The *prrA* mutants show enhanced fitness in the subinhibitory treatment of aminoglycosides. (A) MIC experiment with Kan antibiotic, with concentrations ranging from 0 to 300 μg/mL. The MIC for Kan in both strains is 198 μg/mL, and the subinhibitory concentration is 131 μg/mL. (B) MIC experiment with Strep antibiotic, with concentrations ranging from 0 to 800 μg/mL. The MIC for Kan in both strains is 400 μg/mL, and the subinhibitory concentration is 200 μg/mL. (C) The growth curve of the different strains with the treatment of Kan in the concentration of 100 μg/mL. (D) The growth curve of the different strains with the treatment of Strep in the concentration of 400 μg/mL. (E) Competition assay for WT and prrA mutant coculture. The *y* axis represents the percentage of prrA strain in the different treatments. The above graphs are the average of three independent experiments with three replicates each. Error bars represent the standard deviations.

Further mining of the RNA-seq results showed that members of the *mexXY* efflux pump system genes are significantly upregulated in the antitoxin mutant strain compared to the WT strain (Fig. 5). Since the *mexXY* system in P. aeruginosa is known to affect resistance against aminoglycoside antibiotics (33), we first checked the MIC of the different strains for Kanamycin (Kan) and Streptomycin (Strep) aminoglycosides. The results showed that although the MIC values were not affected by the deletion of *prrA*, in the subinhibitory concentrations, in both examined antibiotics, the mutant showed higher growth compared to the WT strain, while in normal conditions, its growth is significantly inhibited (Fig. 6A and B). To further examine the subinhibitory concentration effect, we performed a growth curve of the different strains with a particular subinhibitory concentration of either Kan or Strep antibiotics. The results showed that the prrA mutant strain began to recover earlier in both antibiotics than the WT and the complementation strains (Fig. 6C and D). We concluded that the PrrA role is presumably important to the recovery from antibiotic inhibition. To further examine the possible fitness advantage this might have, we performed a coculture competition assay between the antitoxin mutant and WT strain grown in the presence of subinhibitory concentrations of aminoglycosides. The results showed that, unlike the control culture in which the percentage of the *prrA* mutant remained roughly 50%, in the treated cocultures, the antitoxin mutant took over and became the primary strain during the growth, highlighting the increased fitness of this strain under these tested conditions (Fig. 6E).

### PrrT/A system is highly distributed among P. aeruginosa strains.

The abundance of the PrrT and PrrA homologs was analyzed in 233 P. aeruginosa genomes (all the existent complete genomes). Although the system was absent from the two laboratory strains PAO1 and PA14, the screen revealed that 132 genomes (56.6%) contained both PrrT and PrrA homologs. Moreover, in all PrrT/A-positive genomes, the PrrT and PrrA homologs were located adjacent to each other, mainly with a short overlap up to 10 bp apart (Table S3). We further investigated whether all of the homolog PrrT/A systems are located close to a prophage by calculating the genomic distance between the PrrT/A homologs and the next prophage. The analysis did not indicate any constant PrrT/A–prophage genomic distance, suggesting that the genomic proximity might not be essential for the PrrT/A function (data not shown).

## DISCUSSION

**FIG 7:**
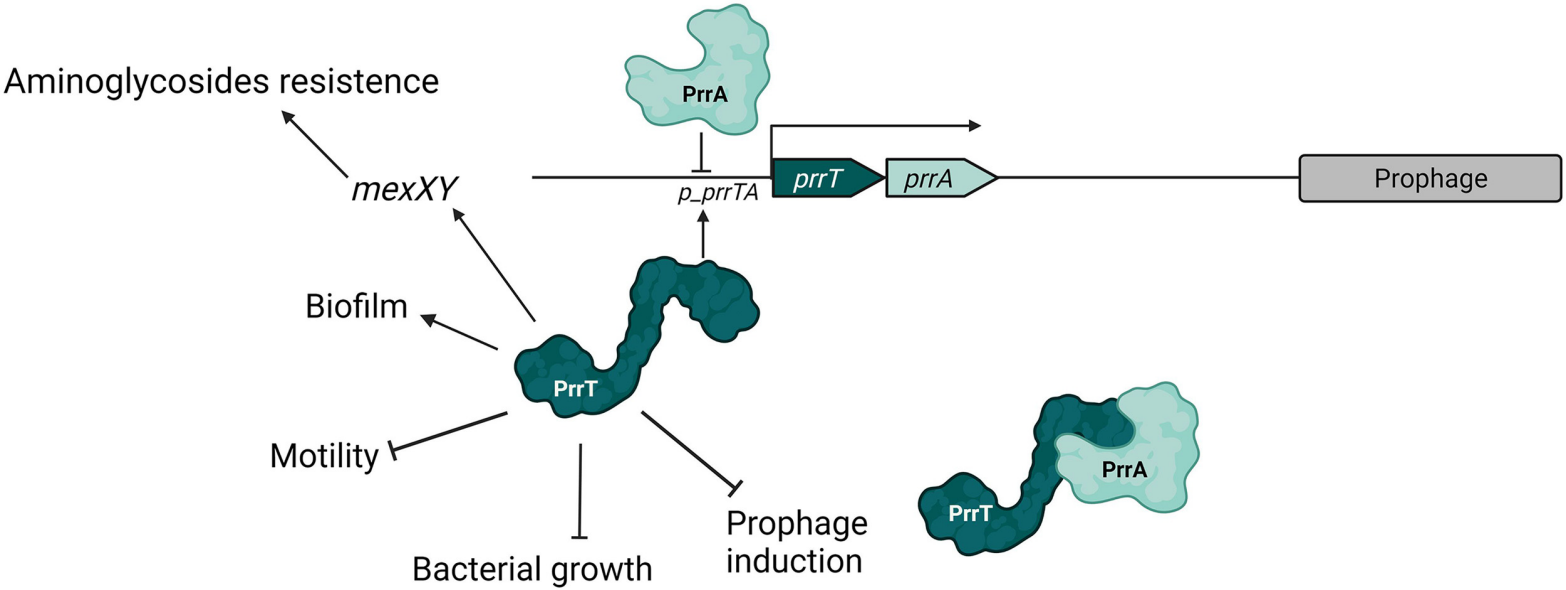
Model for the PrrTA system and its involvement in bacterial processes.

We have identified and characterized a novel type II TA system of P. aeruginosa, which seems like a combined *higA/parE*-like system. We showed that *prrA* and *prrT* are cotranscribed and can interact to form a protein complex together. We examined the system's self-regulation and showed that PrrA antitoxin represses the promoter by direct binding and that PrrT toxin positively regulates the operon expression. We also found that toxin imbalance impacts bacterial growth, increases biofilm formation, reduces motility, and impacts the fitness at subinhibitory concentrations of aminoglycosides. Importantly, we showed that *prrA* deletion led to the repression of expression of prophages' genes over the entire genome, which correlated with reduced phage production. At the same time, *prrA* OE in the host bacteria resulted in decreased phage susceptibility (Fig. 7).

The HigA antitoxin was shown to have an independent promoter apart from the *higA/B* promoter, which results in higher transcript levels of *higA* compared to *higB* toxin in the late stationary phase (24). In the current study, the toxin effect could only be detected by robust and artificial induction in the absence of *prrA*, indicating a significant stoichiometric advantage of PrrA antitoxin. Moreover, the toxin induction also influenced the Δ*prrT* strain, though only in the late stationary phase, suggesting that *prrA*, similarly to *higA*, may have an independent promoter located inside the *prrT* ORF.

In type II TA systems, the antitoxin alone or the TA complex act as a transcriptional auto-repressor (34). The experimental evidence showed that the PrrT-PrrA complex is not required for self-repression as both the *prrT* mutant strain and the *prrT/A* operon mutant with *prrA* induced expression exhibited complete repression with minimum promoter activity. Surprisingly, the PrrT toxin was found to stimulate and increase self-expression in the absence of the PrrA antitoxin. Toxin-driven transcriptional stimulation is mainly attributed to “conditional cooperativity,” a condition in which the repressors are destabilized due to a disruption of the antitoxin/toxin ratio, resulting from toxin excess, allowing resynthesis of both genes (35). Since the PrrT-dependent promoter stimulation was detected in *the prrA* mutant strain, conditional cooperativity poorly accounts for the observed phenomenon. It can only occur if some cross-regulation between other chromosomal TA systems has occurred. An interaction between noncognate complexes of toxins and antitoxins, which can bind to other TA promoter regions and regulate the expression, has been reported (36–38).

The PrrT effect can be attributed to a stress condition as it inhibits bacterial growth. Type II TA systems are influenced by stressors and are thought to influence bacterial survival and tolerance in stress conditions (26). A recently published paper showed that despite the transcriptional increase of the TA genes upon different stress conditions, the toxin is not activated in the examined conditions, and the upregulation is caused solely by antitoxin degradation (26). Consistently, we showed that stress caused by a subinhibitory concentration of NOR resulted in a significant increase in *prrT/A* promoter activity but only in strains carrying an intact *prrA* gene. Notably, NOR and PrrT act similarly; NOR is a fluoroquinolone antibiotic that acts through direct binding to the A subunit of the DNA gyrase (39), and PrrT toxin belongs to ParE family of toxins, which are also gyrase inhibitors that block DNA replication (40). Thus, despite the similar mechanism, the PrrT dependent stimulation effect cannot be attributed to the stress induction as it does not depend on PrrA cleavage.

The deletion of *prrA* antitoxin resulted in increased biofilm formation and reduced motility by elevating DGCs expression, leading to higher bacterial c-di-GMP levels. In contrast, it was shown that the *higA* mutant exhibits reversed phenotype with decreased biofilm formation, elevation in PDEs expression, and reduced c-di-GMP levels (22, 41). The contrasting results are probably attributable to the fact that *prrT/A* is a combined system in which the antitoxin mutant effect is mainly due to the upregulation of the toxin, as the ParE toxin was shown to enhance biofilm formation in E. coli (37) significantly.

Interactions between TA systems and prophages were found and characterized for different TA systems encoded by either the prophage itself (42, 43) or a residual plasmid (44). To our knowledge, no chromosomal type II TA system was described for prophage regulation properties. The deletion of the antitoxin resulted in significant global repression of the prophage's gene expression over the entire genome and decreased phage production. Nearly complete restoration of the phenotype was detected in the double mutant strain, indicating that the toxin upregulation influenced the observed prophage repression. Notably, considering the presumable mechanism of the PrrT toxin, it should have oppositely influenced the phages as it inhibits the gyrase and leads to SOS response activation, and thus the expected outcome would be prophage induction.

Interestingly, a link between phages and AmrZ levels was recently described (45). It has been shown that AmrZ represses CRISPR-Cas immunity genes upon surface attachment and that some phages of Pseudomonas carry *amrZ* homologs to avoid CRISPR defense. Here, we have demonstrated that upon antitoxin deletion, in addition to “prophages” genes’ downregulation and decreased phage induction, *amrZ* is also repressed, which correlates with the published anti-defense properties of *amrZ*, although the 39016 strain does not harbor a CRISPR system.

The OE of *prrA*-antitoxin in the PA14 strain resulted in lower susceptibility to PR5 phages produced by the 39016 strain. TA systems can compensate for each other by cross-reactivity. Therefore, single toxins or antitoxins can still influence the bacteria by reacting with other chromosomal or plasmid-encoded antitoxins or toxins (36, 37). Considering this cross-reactivity ability of TA systems, a reasonable assumption would be some toxin downregulation in the *prrA* OE strain due to an interaction with either a bacterial (PA14) or phage-encoded toxin.

Antibiotic tolerance driven by type II TA systems is mainly associated with the formation of persistence cells (46) or the maintenance of plasmids and genomic islands carrying antibiotic resistance genes (47). The activation of the *mexXY* efflux pump by toxin upregulation has not been described before for type II or other TA classes. As the *mexXY* pumps are not associated with biofilm resistance in P. aeruginosa (48), the enhanced fitness for aminoglycosides observed in the antitoxin mutant is presumably a direct effect of the PrrT toxin.

Although first identified and characterized in a single clinical isolate of P. aeruginosa, the PrrT/A system is highly distributed among the P. aeruginosa strains, indicating its high importance and the significance of the observed characteristics. The described functions of the PrrT/A system, especially the novel prophage regulation function, can significantly contribute to the developing research and knowledge about the chromosomal type II TA systems, their functions, and host contribution.

## MATERIALS AND METHODS

### Bacterial strains, plasmids, and growth media.

The bacterial strains and plasmids used in this study are listed in Table S1. Primers used in this study are listed in Table S2. All strains were grown in LB (Luria-Bertani broth, Difco) at 37°C unless otherwise specified. For the deletion mutants, the following media were used; Vogel Bonner Minimal Medium (VBMM) (49), Pseudomonas Isolation Agar (PIA, Difco), and No Salt Luria-Bertani (NSLB) + 10% sucrose. For DH5α heat shock, BHI (brain heart infusion broth, Difco) media was used. All strains were grown at 37°C unless otherwise specified. Antibiotic concentrations used in this study were 300 μg/mL Carbenicillin (Crb) and 50 μg/mL Gentamicin (Gm) for P. aeruginosa, and 100 μg/mL Ampicillin (Amp) and 30 μg/mL Gm for Escherichia coli.

### DNA manipulation and plasmid construction.

The genomic extraction was performed using the DNeasy Blood & Cell Culture DNA Kit (Qiagen). For DNA fragment amplification, Phusion High-Fidelity DNA polymerase (Thermo) was used. For gene overexpression, primers were designed to complement the beginning and end of each gene, with the addition of either enzyme restriction sites for ligation or an overlap sequence for Gibson assembly. The amplified inserts were purified using NucleoSpin Gel and PCR Clean-Up (Macherey-Nagel). For the ligation assay, inserts and plasmids were digested using the appropriate fast digest restricted enzymes (Thermo). Ligation was conducted using Biogase Fast Ligation Kit (Bio-Lab Ltd.). For the Gibson assembly, inserts were incubated in the appropriate concentration with a linearized plasmid and 2× LigON mixture (EURx). For plasmid extraction, the QIAprep Spin Miniprep Kit (Qiagen) was used. For verification of successful plasmid transformations, the DNA polymerase ReddyMix PCR Kit and universal primers were used.

### Strain construction.

PAO1 and PA14 strains overexpressing *prrA* and *prrT* were created as described previously (50). For 39016 OE strains, the mini-Tn7 vector was used for genomic expression under an arabinose-induced promoter. The creation of the OE 39016 strains using the mini-Tn7 vector and the mini-CTX vector was performed as previously described (51, 52). Gene deletions were performed by homologous recombination as previously described (49) with minor changes using the *ampR* cassette.

### Growth curve.

LB (2 mL) was inoculated with bacterial strains from frozen stocks and incubated overnight at 37°C with shaking (250 rpm). For the OD measurements, the culture was diluted to 0.005 OD (595 nm) in fresh media and transferred to a 96-well plate, 200 μL in each well. Arabinose was added for gene induction (33.3Mm unless otherwise specified). The plates were incubated for 20 h at 37°C with agitation. Optical density measurements at 595 nm were taken every 30 min using the Synergy 2 Multi-Detection Microplate Reader (BioTek). For the plating efficiency measurements, the culture was diluted to 0.005 OD (595 nm) in fresh LB media to a final volume of 15 mL. Samples of 100 μL were taken at 2 h intervals, and serial dilutions were plated in 5 μL drops on top of an LB plate. The plates were incubated ON, and the appearing colonies were counted for CFU/mL calculation.

### Bacterial two-hybrid (BACTH) assay.

The BACTH assay was conducted as described (53). The coding regions of *prrA* and *prrT* were cloned into pUT18C and pKT25, respectively. The recombinant plasmids were cotransformed into E. coli BTH101 competent cells with selection for kanamycin and ampicillin resistance. Eight different single colonies were then resuspended, and 5 μL were spotted on LB plates supplemented with kanamycin, ampicillin, IPTG (0.5 mM), and X‐gal (40 μg/mL). The colonies grew for 6 days at 30°C. Negative controls were included.

### Protein extraction.

PAO1 strains were inoculated to 2 mL LB with antibiotic selection and grown overnight. Bacteria were then diluted 1:100 into M9+CA medium with L-(+)-Arabinose (33.3 μM) and grown to 0.6 OD (595 nm). From each strain, 1.5 OD (595 nm) of bacteria was taken and centrifuged at 14,000 *g* for 2 min, and the supernatant was removed. The cell pellet was then resuspended in lysis buffer (100 mM NaCl; 5% glycerol; 50 mM Tris PH 7.5) containing Benzonase Endonuclease (Millipore), cOmplete protease inhibitor cocktail (Roche), and Lysozyme (Sigma-Aldrich). Samples were then incubated for 15 min at 30°C with agitation followed by Sonication (90 sec, ON 5 sec, OFF 5 sec, 37% amplitude). The sonicated samples were centrifuged at 20,817 *g* for 10 min, and the upper liquid phase containing the proteins was collected.

### Co-immunoprecipitation with ANTI-FLAG resin (co-IP).

Cell lysate (400 μL) was added to ANTI-FLAG M2 Affinity Gel (Sigma-Aldrich), and lysis buffer was added to a final volume of 1 mL and incubated ON with gentle shaking at 4°C. The cell lysate and resin mix was centrifuged at 5,000 *g* for 30 sec, and the supernatant was removed. The mix was then washed three times with 500 μL of TBS (0.8% NaCl; 20 mM Tris 1M pH 7.4; water). One hundred μL of 3× FLAG peptide (Sigma-Aldrich) solution (150 ng/μL final concentration in TBS) was added to the mix and incubated with gentle shaking for 30 min at 4°C. The mix was then centrifuged at 5,000 *g* for 30 sec, and the supernatant was collected. For Western blot analysis, protein samples were diluted 3:1 with Sample BufferX3 (150 mM Tris-HCl pH = 6.8; 3% β-mercaptoethanol; 6% sodium dodecyl sulfate; 0.3% Bromophenol blue; 30% glycerol; water), incubated at 95°C for 10 min and then centrifuged at 14,000 *g* for 2 min. The samples were then separated on a 20% Tris-Glycine gel and transferred to a nitrocellulose membrane. After blocking with 1% alkali-soluble casein in TBS for His or 5% skim milk in TBS for Flag ON at 4°C, the membrane was incubated for 1 h with anti-His tag antibodies (1:1,000; Merck) and anti- FLAG antibodies (1:2,500; Sigma-Aldrich) separately. Following three washes with Tris-buffered saline with Tween 20 (TBST), the membrane was incubated with goat antimouse (HRP) antibodies (1:2,500; Santa Cruz Biotechnology) for an hour. After an additional three TBST washes, the membrane was developed with an ECL kit.

### Electrophoretic mobility shift assay (EMSA).

EMSA was conducted using the LightShift Chemiluminescent EMSA kit (20148. Thermo Fisher Scientific, MA, USA) according to the manufacturer's protocol. The *prrT/A* promoter region was amplified with biotinylated primers (*prom_biot_F* and *prom_biot_R*). To validate the interaction between the PrrA protein and *prrT/A* promoter, decreasing concentrations of a purified PrrA protein (initial concentration of 10 ng/μL) were mixed with binding buffer (×10), NP-40 (1%), ultrapure water, and 50 fmol of a biotinylated *prrT/A* promoter sequence. To establish the position of an unshifted band in the gel, a mixture with the biotinylated *prrT/A* promoter sequence was prepared but without the PrrA protein. To demonstrate that the band shift observed results from a specific protein-DNA interaction, a competition experiment was performed by first incubating the PrrA protein for 10 min at room temperature with an excess of ×120 unlabeled *prrT/A* promoter sequence (6 pmol) (Lane 3 in Fig. 2D). Following an hour of incubation with the labeled *prrT/A* promoter sequence at room temperature, a loading buffer was added, and the samples were run on a 6% native polyacrylamide gel. The gel was then transferred to a Biodyne B nylon membrane (77016, Thermo Fisher Scientific, MA, USA) for 40 min, and the DNA was cross-linked to the membrane using a hand-held UV lamp with a 254 nm bulb for 10 min. The detection was done by chemiluminescence, according to the manufacturer's protocol.

### mCherry reporter construction and fluorescent measurement.

LB (2 mL) was inoculated with bacterial strains carrying m-Cherry-fused promoter from frozen stocks and incubated overnight at 37°C with shaking (250 rpm). The cultures were diluted to 0.005 OD (595 nm) in fresh media and transferred to a 96-well plate, 200 μL in each well. Arabinose or NOR was added (10 mM and 0.1Mm, respectively). After 14 h of incubation at 37°C with shaking, optical density at 595 nm, fluorescent at an excitation wavelength of 580 nm, and emission wavelength of 610 nm were measured using the Synergy 2 Multi-Detection Microplate Reader (BioTek).

### Static biofilm.

Bacteria were scraped from the LB plate and resuspended in 500 μL PBS, diluted to 0.05 OD (595 nm) in 1 mL M9+CA (with 33.3Mm arabinose if needed). Five replicates of 100 μL of each sample were then transferred to a 96-well plate followed by 24 h incubation at 37°C. The following day, OD was measured using the Synergy 2 Multi-Detection Microplate Reader (BioTek) (595 nm). Planktonic bacteria were washed twice with 200 μL deuterium-depleted water (DDW), followed by the addition of 150 μL crystal violet (CV, Sigma-Aldrich Israel Ltd.) and left for incubation at room temperature (RT) for 15 min. The plate was then rinsed with water to remove crystal violet residues, and 200 μL absolute ethanol was added; the plate was incubated at RT for 15 min. One hundred μL elution from each well was transferred into a new 96-well plate, and the new plate was read in the Synergy 2 Multi-Detection Microplate Reader (BioTek) (OD 595 nm).

### Swarming motility.

For swarming motility assay, 2 mL medium (M9+CA) was inoculated with fresh colony and grown overnight at 37°C with shaking. On the following day, bacteria were diluted at 1:10 in the same medium and incubated for 3 h at 37°C with shaking. Bacteria (2.5 μL) were plated in the middle of a 0.5% agar M9 plate and incubated for 48 h.

### Persistence assay (biphasic death curve).

Persistence assay was performed as previously described (54) with minor changes. Briefly, bacterial cultures were incubated for approximately 16 h, diluted in fresh LB media at 1:10 ratio, and further incubated until reaching the OD of 0.9 at 595 nm. The cultures were then treated with a 10-fold MIC of CIP (1.25 μg/mL); the control cultures for each of the strains were not treated. Samples of 100 μL were taken at the following time points: 1 h, 3 h, 5 h, 18 h, and 24 h. Serial dilutions were plated in 10 μL drops on top of an LB plate. The plates were incubated for 24 h, and the appearing colonies were counted for CFU/mL calculation.

### Phage extraction.

LB (2 mL) was inoculated with bacterial strains incubated overnight. On the next day, the bacteria were diluted 1:50 with medium to a final volume of 4 mL and incubated until reaching OD 0.5 at 595 nm (~2 h). For phage induction, 0.4 μg/mL NOR (sigma) antibiotic was added, and the cultures were incubated for 1 h. Fresh LB was added (1.75 mL), and the cultures were incubated for an additional 1 h. Then, 1 mL of bacteria was centrifuged at 14,000 *g* for 2 min, and 900 μL of the supernatant was filtered using a 0.45 μm filter (Whatman).

### Plaque assay.

LB (2 mL) was inoculated with recipient strain and incubated overnight. On the next day, bacteria were diluted at 1:50 with medium to a final volume of 4 mL and incubated for 1.5 h. Serial dilutions (1:10) of the induced phage stock were made. The diluted phage (100 μL) was added to the recipient bacteria (100 μL) and incubated for 15 min at 37°C. The mix of the phage and bacteria was then transferred into 5 mL heated (50°C) LB with 0.5% agar, then gently mixed and poured onto the surface of a 1.5% agar plate. The plate was incubated overnight until plaques were formed. The PFU/mL values are presented as the mean ± SEM.

### RNA extraction.

The RNA extraction was performed as previously described (50). Bacterial strains were inoculated to LB and grown overnight. Bacteria were then diluted 1:100 into 15 mL M9 and grown to 0.5OD (595 nm). For the induced samples, 0.4 μg/mL NOR antibiotic was added, and all the cultures were incubated for an additional 1 h. Two mL of each sample was taken and incubated for 20 min with 4 mL RNAprotect Bacteria Reagent (Qiagen). After incubation, the bacteria were centrifuged at 3,220 *g* for 20 min and rinsed in Tris-EDTA buffer solution (pH 8; Sigma/fluka) to remove RNAprotect residues. Ninety μg/mL Lysozyme (Roche), 10 μL Proteinase K (Qiagen), and 1 mL warm Tri-reagent 37°C (Sigma) were added to pelleted cells. After 5 min of incubation at 65°C, 200 μL chloroform was added. The solution was centrifuged for 15 min at 20,817 *g*, and the upper liquid phase was transferred into 80% ethanol. The RNA was then extracted using an RNeasy minikit (Qiagen) according to the manufacturer's protocol.

### RNA sequencing.

For RNA sequencing, 2 μg of total RNA was used for the RiboMinus Bacteria Transcriptome isolation kit (Invitrogen). The library was constructed with NEBNext Ultra II Directional RNA Library Prep Kit for Illumina (NEB) according to the manufacturer’s instructions using 30 ng of depleted RNA. The final quality was evaluated by TapeStation High Sensitivity D1000 Assay (Agilent Technologies, CA, USA). Sequencing was performed based on Qubit values and loaded onto an Illumina MiSeq using the MiSeq V2 (50 cycles) Kit (Illumina, CA, USA). Single-end RNA-seq protocol was used, yielding about 1.34–1.74 million reads per sample. FastQC (v0.11.2) (https://www.bioinformatics.babraham.ac.uk/projects/fastqc) was used to assess the quality of raw reads. Reads were aligned to P. aeruginosa 39016 strain (RefSeq sequence ID: NZ_CM001020) using the Bowtie2 (55) aligner software (version bowtie2-2.3.2) with default parameters. GTF annotation file for the 39016 strain wad downloaded from NCBI (https://www.ncbi.nlm.nih.gov/assembly/GCF_000148745.1, downloaded March 2019), and the *prrT*_ORF coordinates were added manually to the annotation file. Raw read counts for 6469 gene-level features were determined using HTSeq-count (56) with the intersection-strict mode. Differentially expressed genes were determined with the R Bioconductor package DESeq2 (57). The *P* values were corrected with Benjamini-Hochberg FDR procedure. Genes with adjusted *P* values < 0.05 and |log fold change| > 1 were considered as differentially expressed.

### Real-time PCR analysis (RT-PCR).

For cDNA production, the GoScript Reverse Transcription System (Promega) was used with 1 μg RNA according to the manufacturer's instructions. For the RT-PCR analysis, Fast SYBR Green Master Mix was used (Applied Biosystems, Thermo), according to the manufacturer's instructions. RT-PCR was conducted using CFX-96 Touch Real-Time PCR Detection System (Bio-Rad). Results were normalized using the PA3540 housekeeping gene.

### MIC-MIC.

LB (2 mL) was inoculated with bacterial strains grown on LB plates and incubated overnight. Double dilutions of the antibiotics were performed. Bacteria were then diluted and transferred to a 96-well plate to a final concentration of 0.001 OD (595 nm). The plates were incubated overnight with shaking. Optical density measurements at 595 nm were taken using Synergy 2 Multi-Detection Microplate Reader (BioTek).

### Competition assay.

LB (2 mL) was inoculated with bacterial strains grown on LB plates and incubated overnight. Initial amount of 10^6^ cells were added from each strain into 15 mL of fresh LB media. The subinhibitory concentrations of the antibiotics were added immediately (except for the control coculture). Samples of 100 μL were taken at the following time points: 0 h, 3 h, 6 h, 24 h. Serial dilutions were plated in 5 μL drops on LB and LB-carbenicillin (CRB) plates. The plates were incubated for ON, and the appearing colonies were counted for CFU/mL calculation. Since the Δ*prrA* strain is the only CRB-resistant strain, its percentage was calculated as the ratio of plating efficiency observed on the LB-CRB to the plating efficiency on the LB plate, representing the overall efficiency.

### PrrA and PrrT orthologs search.

Reciprocal best hit of BLAST (58) was used to find orthologs of *prrA* and *prrT*. The amino-acid sequences of PrrT and PrrA and complete genomes of 233 P. aeruginosa strains were downloaded from Pseudomonas Genome DB version 20.2 (59). First, tblastn (blast package, version 2.5.0 (was used to find matches (in protein level search) of PrrA or PrrT proteins in each of the 233 P. aeruginosa genomes. Then, for verification, for genomes with a match, tblastx was performed on the prrA and/or prrT orthologs (only matches with e-value < 0.05 were considered) against the 39016 genome to verify that it best matches to the PrrA or PrrT.

### General bioinformatics.

Identification of prophage regions and genes was conducted using PHASTER (60) (PHAge Search Tool). Prophage gene identification was achieved using the Pseudomonas genome database (59). Gene comparison was conducted using BLAST (61). Gene annotation was performed using RAST (7).

### Data availability.

The data that support the findings of this study are available in the GEO database (accession number GSE179116).
