# Supplementary material for: PrrT/A, a Pseudomonas aeruginosa Bacterial Encoded Toxin-Antitoxin System Involved in Prophage Regulation and Biofilm Formation
Source: Microbiol Spectr. 2022 May 16;10(3):e01182-22. doi: 10.1128/spectrum.01182-22 (PMC9241795; doi:10.1128/spectrum.01182-22)
Supplement: SUPPLEMENTAL FILE 1 — Supplemental material. Download spectrum.01182-22-s001.pdf, PDF file, 1 MB [file spectrum.01182-22-s001.pdf]

Supplementary materials

Table S1

Strain or plasmid	Description	Source
	***P. aeruginosa* strains**	
PA01	Wild-type	(1)
PAO1/ *prrA*-FLAG	Wild type with pUCP18/prrA_FLAG plasmid,	This study
PAO1/ *prrT*-HIS	Wild type with pJN105/prrT_HIS plasmid, Gm^r	This study
PAO1/ *prrT*-HIS/*prrA*-FLAG	Wild type with pJN105/prrT_HIS and prrT_HIS plasmids, Gm^r, crb^r	This study
39016	LMG 27,647 *P. aeruginosa* (Schoeter 1872) migula 1900 AL	BCCM- biological origin: keratitis patients
39016 Δ*prrA*	39016 with *ampR* marked deletion of *prrA*, crb^r	This study
39016 Δ*prrT*	39016 with *ampR* marked deletion of *prrT*, crb^r	This study
39016 Δ*prrTA*	39016 with *ampR* marked deletion of *prrT*/*prrA*, crb^r	This study
39016 Δ*prrA*/*prrA*	39016 with *ampR* marked deletion of *prrA*, with *att-tn7* insertion of arabinose inducible *prrA*, Gm^r crb^r	This study
39016 Δ*prrA*/*prrT*	39016 Δ*prrA*, with *att-tn7* insertion of arabinose inducible *prrA*, Gm^r crb^r	This study
39016 Δ*prrT*/*prrT*	39016 Δ*prrT*, with *att-tn7* insertion of arabinose inducible *prrA*, Gm^r crb^r	This study
39016 Δ*prrTA*/*prrT*	39016 Δ*prrTA*, with *att-tn7* insertion of arabinose inducible *prrA*, Gm^r crb^r	This study
39016 /mCherry	39016 with *att-ctx* insertion of *prrT*/*A* promoter fused to mCherry	This study
39016 Δ*prrA*/mCherry	39016 Δ*prrA* with *att-ctx* insertion of *prrT*/*A* promoter fused to mCherry	This study
39016 Δ*prrT*/mCherry	39016 Δ*prrT* with *att-ctx* insertion of *prrT*/*A* promoter fused to mCherry	This study
39016 Δ*prrTA*/mCherry	39016 Δ*prrTA* with *att-ctx* insertion of *prrT*/*A* promoter fused to mCherry	This study
39016 Δ*prrTA*/*prrT*/mCherry	39016 Δ*prrTA*/*prrT* with *att-ctx* insertion of *prrT*/*A* promoter fused to mCherry	This study
39016 Δ*prrTA*/*prrA*/mCherry	39016 Δ*prrTA*/*prrA* with *att-ctx* insertion of *prrT*/*A* promoter fused to mCherry	This study
PA14	Wild-type	(2)
PA14/ *prrA*	PA14 with pUCP18/prrA plasmid, crb^r	This study
PA14/ VEC	PA14 with pUCP18 plasmid, crb^r	This study
	***E. coli* strains**	
DH5*α*	F^- Φ80lacZΔM15 Δ(lacZYA-argF) U169 recA1 endA1 hsdR17 (rK-, mK+) phoA supE44 *λ*- thi-1 gyrA96 relA1.	Bio-Lab
S17	*E. coli* S17 thi, pro, hsdR, recA::RP4 -2-Tc::Mu aphA::Tn7, *λ*-pir, Smr , Tpr	(3)
BTH101	F^- , cya-99, araD139, galE15, galK16, rpsL1 (Str^r), hsdR2, mcrA1, mcrB1	(4)
BTH101 *prrA*	BTH101 with pUT18C/prrA and an empty pKT25 plasmids, amp^r, kan^r, str^r	This study

BTH101 *prrT*	BTH101 with pKT25/prrT and an empty pUT18C plasmids, amp^r, kan^r, str^r	This study
BTH101 *prrA/prrT*	BTH101 with pKT25/prrT and pUT18C/prrA plasmids, amp^r, kan^r, str^r	This study
BTH101 vec	BTH101 with empty pUT18C and pKT25 plasmids, amp^r, kan^r, str^r	This study
**Plasmids**		
pUCP18-Ap	Crb^r (for *P. aeruginosa*), Amp^r (for *E. coli*), overexpression plasmid, *lacZ* promoter	(5)
pDONRPEX18Gm	Gm^r and Cm^r, pEX18Gm containing a HindIII flanked, *attP* cloning site from pDONR201	(6)
mini-CTX-LacZ	TetR, integration vector for single-copy, chromosomal lacZ fusions; Ω-FRT-attP-MCS, ori, int, and oriT	(7)
pUCP18T-miniTN7T-Gm	Amp^r and Gm^r; Gmr on mini-Tn7T, mobilizable suicide plasmid	(8)
pJN105	Overexpression plasmid under araC prmoter	(9)
pKT25	Kan^r pSU40 derivative plasmid, encodes for T25 fragments under lac promoter. For expression of chimeric protein fused to the C-terminal end of T18.	(4)
pUT18C	Amp^r pUC19 derivative plasmid, encodes for T18 fragments under lac promoter. For expression of chimeric protein fused to the C-terminal end of T18.	(4)

Table S2

Primer	Sequence
**M13_F**	CCCAGTCACGACGTTGTAAAACG
**M13_R**	AGCGGATAACAATTTCACACAGG
**AmpR_F**	CGCGGAACCCCTATTTGTT
**AmpR_R**	TTACCAATGCTTAATCAGTGAGG
**prrA _ko_upF_GWB1**	GGGGACAAGTTTGTACAAAAAAGCAGGCTCAGGAATGAGGCTGCCTCGC
**prrA_ampR_ko_upR**	AACAAATAGGGGTTCCGCGCGATCAGTCCCTCCTCACG
**prrA_ampR_ko_DownF**	CCTCACTGATTAAGCATTGGTAAGGAAGCTCCGAACAAGTCAG
**prrA _ko_DownR_GWB2**	GGGGACCACTTTGTACAAGAAAGCTGGGTAGTCGGTGGGCGGACTCAA
**prrT_ko_upF**	ACATCAGAGATTTTGAGACACGGGCCAGAGCGCAATGCAACCAGCATCC
**prrT_ampR_ko_upR**	TTAGAAAAATAAACAAATAGGGGTTCCGCGAAAATATAACCCCCAGGGAATATT
**prrT_ampR_ko_DownF**	ATAGGTGCCTCACTGATTAAGCATTGGTAAATGGTTAAGAAATTCTCCGACCT
**prrT_ko_DownR**	GCAAGCTTGTTAACGCTAGCATGGATCTCGTGACCAAGAACACCGTGCAA
**prrA_F_pUCP18**	ATCCAGAGCTCTAAGGAGGAATAACATTCGATGGTTAAGAAATTCTCCGAC
**prrA_R_pUCP18**	ATCCAAAGCTTTTAGGCGCCCGCCTCTTC
**prrA_R_FLAG_pUCP18**	ATCCAAAGCTTTTACTTGTCATCGTCATCCTTGTAGTCGGCGCCTGCCTCTTCGAT

**prrT_F_pJN105**	ATCCATCTAGACTTCTGAATAAGCTTCTGTGGCGTGGGATATCGAGT
**prrT_R_pJN105**	ATCCAGAGCTCTTAACCATCGATCAGTCCCTC
**prrT_R_HIS_pJN105**	ATCCAGAGCTCTTAGTGATGATGATGATGATGACCATCGATCAGTCCCTCC
**prrA_F_miniTn7**	ATCCATCTAGATAAGGAGGAATAACATTCGATGGTTAAGAAATTCTCCGAC
**prrA_R_miniTn7**	ATCCAGAGCTCTTAGGCGCCCGCCTCTTC
**araC_F**	GGGCCCCCAATTATGACAACTTGACGGC
**prrT/A_Prom_F_miniCtx**	TTACGCGAAATACGGGCAGACATGGCCTGCCCGGTTATTAACCGATAACCGACGGAGGC
**prrT/A_Prom_R_mCherry**	TCCTCCTCGCCCTTGCTCACAAAATATAACCCCCAGGGAATAT
**mCherry_F_prrT/A_prom**	TTCCCTGGGGGTTATATTTTGTGAGCAAGGGCGAGGA
**mCherry_R_miniCtx**	TCATCCACCGGCGCGCGTAATACGACTCACTATAGGGCGATACTTGTACAGCTCGTCCATG
**Prom_biot_F**	5'BIOT/GACTGGGGCTGGATCGGA
**Prom_biot_R**	5'BIOT/AAAATATAACCCCCAGGGAATATT
**amrZ_F_qPCR**	CAAATTCGTCGTTCGTCTGC
**amrZ_R_qPCR**	CACCGAGATTGTCTTGCAGC
**siaD_F_qPCR**	AAGAAGTCCACGTCGAGCA
**siaD_R_qPCR**	TGAAGGAAGCCTCGATCCG
**morA_F_qPCR**	GATCAGCCTGTTTTCCGAGC
**morA_R_qPCR**	GAACTGGAAGGTATTGCCGC
**nbdA_F_qPCR**	TGCTGAAGATCGTCTCCGAA
**nbdA_R_qPCR**	ATCTTCTGCATCAGGCGTTG
**PA3540_F_qPCR**	TGTCGCGCTACTACATGCGTC
**PA3540_R_qPCR**	GTGTCGTGGCTGGTGATGAGA
**prrA_F_pUT18C**	CCGGATTGCGGCGGCCGTCGCTGGGCGCAGTGGAACGCCAGTTAAGAAATTCTCCGACCT TCG
**prrA_R_pUT18C**	GAGCAGATTGTACTGAGAGTGCACCATATTACTTAGTTATGGCGCCCGCCTCTTCGA
**prrT_F_pKT25**	CCGATTACCTGGCGCGCACGCGGCGGGCTGCAGGGTCGACGCGTGGGATATCGAGTACAC
**prrT_R_pKT25**	CACGACGTTGTAAAACGACGGCCGAATTCTTAGTTACTTAACCATCGATCAGTCCCTCCT

Table S3

Genome	***prrA*** **loci**	***prrT*** **loci**	***prrA-prrT*** **(bp)[a]**
Pseudomonas_aeruginosa _12-4-4_59_3618	NA	NA	NA

Pseudomonas_aeruginosa_12939_6590	NA	NA	NA
Pseudomonas_aeruginosa_1334_14_10731	NA	NA	NA
Pseudomonas_aeruginosa_1811-13R031_12048	NZ_CP046061.1:chromosome:5382108-5382431	NZ_CP046061.1:chromosome:5382424-5382789	0
Pseudomonas_aeruginosa_1811-18R001_12049	NZ_CP046060.1:chromosome:5382312-5382635	NZ_CP046060.1:chromosome:5382628-5382993	0
Pseudomonas_aeruginosa_19BR_362	NZ_AFXJ01000001.1:chromosome:901583-901906	NZ_AFXJ01000001.1:chromosome:901225-901590	0
Pseudomonas_aeruginosa_213BR_361	NZ_AFXK01000001.1:chromosome:901762-902085	NZ_AFXK01000001.1:chromosome:901404-901769	0
Pseudomonas_aeruginosa_243931_11950	NZ_CP041772.1:chromosome:677501-677824	NZ_CP041772.1:chromosome:677817-678182	0
Pseudomonas_aeruginosa_24Pae112_9634	NZ_CP029605.1:chromosome:5140503-5140826	NZ_CP029605.1:chromosome:5140819-5141184	0
Pseudomonas_aeruginosa_268_9897	NZ_CP032761.1:chromosome:5325042-5325365	NZ_CP032761.1:chromosome:5325358-5325723	0
Pseudomonas_aeruginosa_519119_11949	NZ_CP041773.1:chromosome:713919-714242	NZ_CP041773.1:chromosome:714235-714600	0
Pseudomonas_aeruginosa_60503_11947	NZ_CP041774.1:chromosome:4980008-4980331	NZ_CP041774.1:chromosome:4980341-4980688	10
Pseudomonas_aeruginosa_8380_3922	NZ_AP014839.1:chromosome:4843947-4844270	NZ_AP014839.1:chromosome:4844263-4844628	0
Pseudomonas_aeruginosa_97_6674	NA	NA	NA
Pseudomonas_aeruginosa_97_9520	NA	NA	NA
Pseudomonas_aeruginosa_A681_11948	NA	NA	NA
Pseudomonas_aeruginosa_AES1M_10829	NZ_CP037925.1:chromosome:1357130-1357453	NZ_CP037925.1:chromosome:1356772-1357137	0
Pseudomonas_aeruginosa_AES1R_10830	NZ_CP037926.1:chromosome:4720087-4720410	NZ_CP037926.1:chromosome:4720403-4720768	0
Pseudomonas_aeruginosa_AG1_12046	NZ_CP045739.1:chromosome:5298704-5299027	NZ_CP045739.1:chromosome:5299020-5299367	0
Pseudomonas_aeruginosa_AR_0095_6299	NZ_CP027538.1:chromosome:4732527-4732850	NZ_CP027538.1:chromosome:4732169-4732534	0
Pseudomonas_aeruginosa_AR_0110_6371	NA	NA	NA
Pseudomonas_aeruginosa_AR_0111_9636	NA	NA	NA
Pseudomonas_aeruginosa_AR_0230_6281	NA	NA	NA

Pseudomonas_aeruginosa_AR_0353_6205	NZ_CP027172.1:chromosome:1917031-1917354	NZ_CP027172.1:chromosome:1916673-1917038	0
Pseudomonas_aeruginosa_AR_0354_6975	NZ_CP027171.1:chromosome:2827871-2828194	NZ_CP027171.1:chromosome:2828187-2828552	0
Pseudomonas_aeruginosa_AR_0356_7083	NA	NA	NA
Pseudomonas_aeruginosa_AR_0357_7058	NZ_CP027166.1:chromosome:3591865-3592188	NZ_CP027166.1:chromosome:3591507-3591872	0
Pseudomonas_aeruginosa_AR_0360_6841	NA	NA	NA
Pseudomonas_aeruginosa_AR_0446_6897	NA	NA	NA
Pseudomonas_aeruginosa_AR439_6890	NA	NA	NA
Pseudomonas_aeruginosa_AR441_6528	NA	NA	NA
Pseudomonas_aeruginosa_AR442_6465	NZ_CP029090.1:chromosome:4419525-4419848	NZ_CP029090.1:chromosome:4419841-4420206	0
Pseudomonas_aeruginosa_AR444_6466	NA	NA	NA
Pseudomonas_aeruginosa_AR445_6560	NZ_CP029088.1:chromosome:5234080-5234403	NZ_CP029088.1:chromosome:5234396-5234743	0
Pseudomonas_aeruginosa_AR_455_7148	NA	NA	NA
Pseudomonas_aeruginosa_AR_458_7202	NA	NA	NA
Pseudomonas_aeruginosa_AR_460_7261	NA	NA	NA
Pseudomonas_aeruginosa_ATCC_15692_4007	NA	NA	NA
Pseudomonas_aeruginosa_ATCC_27853_3989	NA	NA	NA
Pseudomonas_aeruginosa_ATCC_27853_9518	NA	NA	NA
Pseudomonas_aeruginosa_AZPAE15042_11928	NA	NA	NA
Pseudomonas_aeruginosa_B136-33_191	NC_020912.1:chromosome:4662648-4662971	NC_020912.1:chromosome:4662964-4663329	0
Pseudomonas_aeruginosa_B14130_10394	NA	NA	NA
Pseudomonas_aeruginosa_B17932_10395	NA	NA	NA
Pseudomonas_aeruginosa_B41226_10391	NA	NA	NA
Pseudomonas_aeruginosa_BA15561_9944	NA	NA	NA
Pseudomonas_aeruginosa_BA7823_9659	NA	NA	NA
Pseudomonas_aeruginosa_BAMC_07-48_3994	NZ_CP015377.1:chromosome:229714-230037	NZ_CP015377.1:chromosome:230030-230395	0
Pseudomonas_aeruginosa_C79_11954	NA	NA	NA

Pseudomonas_aeruginosa_Carb01_63_2626	NZ_CP011317.1:chromosome:5425890-5426213	NZ_CP011317.1:chromosome:5426206-5426553	0
Pseudomonas_aeruginosa_CCUG_51971_11974	NZ_CP043328.1:chromosome:5093302-5093625	NZ_CP043328.1:chromosome:5093618-5093983	0
Pseudomonas_aeruginosa_CCUG_70744_6254	NZ_CP023255.1:chromosome:3020126-3020449	NZ_CP023255.1:chromosome:3020442-3020807	0
Pseudomonas_aeruginosa_CFSAN084950_12043	NZ_CP045768.1:chromosome:3723230-3723553	NZ_CP045768.1:chromosome:3723546-3723911	0
Pseudomonas_aeruginosa_CR1_6461	NA	NA	NA
Pseudomonas_aeruginosa_CR1_7655	NA	NA	NA
Pseudomonas_aeruginosa_DH01_3915	NZ_CP013993.1:chromosome:5265765-5266088	NZ_CP013993.1:chromosome:5266081-5266446	0
Pseudomonas_aeruginosa_DK1_substr_NH57388A_6643	NA	NA	NA
Pseudomonas_aeruginosa_DK2_174	NA	NA	NA
Pseudomonas_aeruginosa_DN1_5583	NA	NA	NA
Pseudomonas_aeruginosa_DN1_7646	NA	NA	NA
Pseudomonas_aeruginosa_DSM_50071_2857	NZ_CP012001.1:chromosome:4623083-4623406	NZ_CP012001.1:chromosome:4623399-4623764	0
Pseudomonas_aeruginosa_E6130952_7221	NZ_CP020603.1:chromosome:5201097-5201420	NZ_CP020603.1:chromosome:5201413-5201778	0
Pseudomonas_aeruginosa_E80_10733	NZ_CP031677.1:chromosome:5090735-5091058	NZ_CP031677.1:chromosome:5091051-5091416	0
Pseudomonas_aeruginosa_E90_12001	NZ_CP044006.1:chromosome:5078878-5079201	NZ_CP044006.1:chromosome:5079194-5079559	0
Pseudomonas_aeruginosa_F22031_3096	NA	NA	NA
Pseudomonas_aeruginosa_F23197_6110	NZ_CP008856.2:chromosome:4764867-4765190	NZ_CP008856.2:chromosome:4765183-4765548	0
Pseudomonas_aeruginosa_F23197_7640	NZ_CP008856.1:chromosome:4605794-4606117	NZ_CP008856.1:chromosome:4605436-4605801	0
Pseudomonas_aeruginosa_F5677_6730	NZ_CP026680.1:chromosome:4823788-4824111	NZ_CP026680.1:chromosome:4824104-4824451	0
Pseudomonas_aeruginosa_F63912_7033	NZ_CP008858.2:chromosome:4857393-4857716	NZ_CP008858.2:chromosome:4857709-4858074	0
Pseudomonas_aeruginosa_F63912_7645	NZ_CP008858.1:chromosome:471683-472006	NZ_CP008858.1:chromosome:471325-471690	0
Pseudomonas_aeruginosa_F9676_2900	NZ_CP012066.1:chromosome:1235086-1235409	NZ_CP012066.1:chromosome:1234728-1235093	0

Pseudomonas_aeruginosa_FA-HZ1_4011	NZ_CP017353.1:chromosome:861431-861754	NZ_CP017353.1:chromosome:861747-862112	0
Pseudomonas_aeruginosa_FDAARGOS_501_9954	NZ_CP033843.1:chromosome:743127-743450	NZ_CP033843.1:chromosome:743443-743808	0
Pseudomonas_aeruginosa_FDAARGOS_505_9955	NA	NA	NA
Pseudomonas_aeruginosa_FDAARGOS_532_9952	NA	NA	NA
Pseudomonas_aeruginosa_FDAARGOS_570_9956	NZ_CP033835.1:chromosome:1982020-1982343	NZ_CP033835.1:chromosome:1981662-1982027	0
Pseudomonas_aeruginosa_FDAARGOS_571_9953	NZ_CP033833.1:chromosome:1390474-1390797	NZ_CP033833.1:chromosome:1390790-1391155	0
Pseudomonas_aeruginosa_FDAARGOS_610_11151	NZ_CP041013.1:chromosome:1832789-1833112	NZ_CP041013.1:chromosome:1832431-1832796	0
Pseudomonas_aeruginosa_FDAARGOS_767_11150	NA	NA	NA
Pseudomonas_aeruginosa_FRD1_2621	NZ_CP010555.1:chromosome:6028298-6028621	NZ_CP010555.1:chromosome:6027958-6028305	0
Pseudomonas_aeruginosa_GIMC5015:PAKB6_10430	NA	NA	NA
Pseudomonas_aeruginosa_H25883_9950	NA	NA	NA
Pseudomonas_aeruginosa_H26023_9951	NA	NA	NA
Pseudomonas_aeruginosa_H26027_9949	NA	NA	NA
Pseudomonas_aeruginosa_H27930_5910	NA	NA	NA
Pseudomonas_aeruginosa_H27930_7642	NA	NA	NA
Pseudomonas_aeruginosa_H5708_6745	NZ_CP008859.2:chromosome:4674110-4674433	NZ_CP008859.2:chromosome:4674426-4674791	0
Pseudomonas_aeruginosa_H5708_7641	NZ_CP008859.1:chromosome:2428869-2429192	NZ_CP008859.1:chromosome:2428511-2428876	0
Pseudomonas_aeruginosa_HOU1_11979	NZ_CP042269.1:chromosome:4486040-4486363	NZ_CP042269.1:chromosome:4486356-4486721	0
Pseudomonas_aeruginosa_HS9_6770	NZ_CP030861.1:chromosome:4373149-4373472	NZ_CP030861.1:chromosome:4372791-4373156	0
Pseudomonas_aeruginosa_IMP-13_10390	NA	NA	NA
Pseudomonas_aeruginosa_IMP66_11969	NZ_CP028959.1:chromosome:4684351-4684674	NZ_CP028959.1:chromosome:4684667-4685032	0
Pseudomonas_aeruginosa_IMP67_11967	NZ_CP028848.1:chromosome:4671378-4671701	NZ_CP028848.1:chromosome:4671694-4672059	0
Pseudomonas_aeruginosa_IMP68_11968	NZ_CP028849.1:chromosome:4669435-4669758	NZ_CP028849.1:chromosome:4669751-4670116	0

Pseudomonas_aeruginosa_INP-43_12123	NA	NA	NA
Pseudomonas_aeruginosa_IOMTU_133_3923	NZ_AP017302.1:chromosome:5084451-5084774	NZ_AP017302.1:chromosome:5084767-5085132	0
Pseudomonas_aeruginosa_isolate_1_11152	NZ_LS998783.1:gnlDSMZPA5486Chr:5268691-5269014	NZ_LS998783.1:gnlDSMZPA5486Chr:5269007-5269354	0
Pseudomonas_aeruginosa_isolate_B10W_5627	NZ_CP017969.1:chromosome:4803304-4803627	NZ_CP017969.1:chromosome:4803620-4803985	0
Pseudomonas_aeruginosa_isolate_early_isolate_NN2_clone_C_6774	NA	NA	NA
Pseudomonas_aeruginosa_isolate_F30658_3634	NZ_CP008857.1:chromosome:2598431-2598754	NZ_CP008857.1:chromosome:2598747-2599094	0
Pseudomonas_aeruginosa_isolate_F9670_3848	NA	NA	NA
Pseudomonas_aeruginosa_isolate_H47921_3637	NA	NA	NA
Pseudomonas_aeruginosa_isolate_M37351_3639	NA	NA	NA
Pseudomonas_aeruginosa_isolate_PA14Or_reads_4013	NA	NA	NA
Pseudomonas_aeruginosa_isolate_paerg000_11157	NZ_LR130528.1:paerg000-contig000:2179667-2179990	NZ_LR130528.1:paerg000-contig000:2179309-2179674	0
Pseudomonas_aeruginosa_isolate_paerg002_11158	NZ_LR130527.1:paerg002-contig000:1276671-1276994	NZ_LR130527.1:paerg002-contig000:1276987-1277352	0
Pseudomonas_aeruginosa_isolate_paerg003_11160	NZ_LR130530.1:paerg003-contig000:4595901-4596224	NZ_LR130530.1:paerg003-contig000:4596217-4596582	0
Pseudomonas_aeruginosa_isolate_paerg004_11159	NZ_LR130531.1:paerg004-contig000:1913627-1913950	NZ_LR130531.1:paerg004-contig000:1913943-1914308	0
Pseudomonas_aeruginosa_isolate_paerg005_11161	NA	NA	NA
Pseudomonas_aeruginosa_isolate_paerg009_11162	NA	NA	NA
Pseudomonas_aeruginosa_isolate_paerg010_11163	NZ_LR130536.1:paerg010-contig000:4595890-4596213	NZ_LR130536.1:paerg010-contig000:4596206-4596571	0
Pseudomonas_aeruginosa_isolate_paerg011_11164	NZ_LR130535.1:paerg011-contig000:4596034-4596357	NZ_LR130535.1:paerg011-contig000:4596350-4596715	0
Pseudomonas_aeruginosa_isolate_paerg012_11165	NZ_LR130537.1:paerg012-contig000:4595911-4596234	NZ_LR130537.1:paerg012-contig000:4596227-4596592	0

Pseudomonas_aeruginosa_isolate_PcyII-10_5293	NZ_LT673656.1:chromosome:4613920-4614243	NZ_LT673656.1:chromosome:4614236-4614601	0
Pseudomonas_aeruginosa_isolate_RW109_6944	NZ_LT969520.1:chromosome:5105589-5105912	NZ_LT969520.1:chromosome:5105905-5106252	0
Pseudomonas_aeruginosa_isolate_T52373_3627	NZ_CP008867.1:chromosome:826684-827007	NZ_CP008867.1:chromosome:827000-827365	0
Pseudomonas_aeruginosa_isolate_T63266_3628	NZ_CP008868.1:chromosome:28734-29057	NZ_CP008868.1:chromosome:29050-29415	0
Pseudomonas_aeruginosa_JB2_7002	NA	NA	NA
Pseudomonas_aeruginosa_K34-7_7082	NA	NA	NA
Pseudomonas_aeruginosa_KRP1_12050	NZ_CP046069.1:chromosome:4767417-4767740	NZ_CP046069.1:chromosome:4767733-4768098	0
Pseudomonas_aeruginosa_L10_6318	NA	NA	NA
Pseudomonas_aeruginosa_LES431_489	NC_023066.1:chromosome:4918112-4918435	NC_023066.1:chromosome:4918428-4918793	0
Pseudomonas_aeruginosa_LESB58_125	NC_011770:chromosome:4970871-4971194	NC_011770:chromosome:4971187-4971552	0
Pseudomonas_aeruginosa_LW_10696	NZ_CP022478.1:chromosome:3792962-3793285	NZ_CP022478.1:chromosome:3793278-3793643	0
Pseudomonas_aeruginosa_M1608_7239	NA	NA	NA
Pseudomonas_aeruginosa_M1608_7643	NA	NA	NA
Pseudomonas_aeruginosa_M18_172	NC_017548.1:chromosome:4658643-4658966	NC_017548.1:chromosome:4658959-4659324	0
Pseudomonas_aeruginosa_MRSN12280_6456	NZ_CP028162.1:chromosome:5195357-5195680	NZ_CP028162.1:chromosome:5195673-5196038	0
Pseudomonas_aeruginosa_MTB-1_210	NC_023019.1:chromosome:4727093-4727416	NC_023019.1:chromosome:4727426-4727773	10
Pseudomonas_aeruginosa_N15-01092_9635	NZ_CP012901.1:chromosome:1762355-1762678	NZ_CP012901.1:chromosome:1762015-1762362	0
Pseudomonas_aeruginosa_N17-1_3988	NZ_CP014948.1:chromosome:4732454-4732777	NZ_CP014948.1:chromosome:4732770-4733135	0
Pseudomonas_aeruginosa_NCGM1900_2620	NZ_AP014622.1:chromosome:2005967-2006290	NZ_AP014622.1:chromosome:2006283-2006648	0
Pseudomonas_aeruginosa_NCGM1984_2619	NZ_AP014646.1:chromosome:5023534-5023857	NZ_AP014646.1:chromosome:5023850-5024215	0
Pseudomonas_aeruginosa_NCGM257_3921	NA	NA	NA
Pseudomonas_aeruginosa_NCGM2S1_173	NC_017549.1:chromosome:1696665-1696988	NC_017549.1:chromosome:1696307-1696672	0

Pseudomonas_aeruginosa_NCTC10728_11169	NZ_LR134342.1:ERS1178933SCcontig000001:1320368-1320691	NZ_LR134342.1:ERS1178933SCcontig000001:1320684-1321049	0
Pseudomonas_aeruginosa_NCTC11445_11166	NZ_LR134308.1:ERS1043809SCcontig000001:503114-503437	NZ_LR134308.1:ERS1043809SCcontig000001:502756-503121	0
Pseudomonas_aeruginosa_NCTC12903_11167	NA	NA	NA
Pseudomonas_aeruginosa_NCTC13359_11326	NZ_LR590473.1:ERS666351SCcontig000001:5395570-5395893	NZ_LR590473.1:ERS666351SCcontig000001:5395886-5396251	0
Pseudomonas_aeruginosa_NCTC13618_11327	NZ_LR590474.1:ERS666353SCcontig000001:4951694-4952017	NZ_LR590474.1:ERS666353SCcontig000001:4952010-4952375	0
Pseudomonas_aeruginosa_NCTC13620_12150	NA	NA	NA
Pseudomonas_aeruginosa_NCTC13715_11168	NZ_LR134330.1:ERS1110714SCcontig000001:47137-47460	NZ_LR134330.1:ERS1110714SCcontig000001:47470-47817	10
Pseudomonas_aeruginosa_NHmuc_5737	NA	NA	NA
Pseudomonas_aeruginosa_Ocean-1155_5897	NA	NA	NA
Pseudomonas_aeruginosa_Ocean-1175_6075	NA	NA	NA
Pseudomonas_aeruginosa_PA1088_5724	NZ_CP015001.1:chromosome:4914519-4914842	NZ_CP015001.1:chromosome:4914835-4915200	0
Pseudomonas_aeruginosa_PA11803_5097	NZ_CP015003.1:chromosome:5211016-5211339	NZ_CP015003.1:chromosome:5211332-5211697	0
Pseudomonas_aeruginosa_Pa1207_5865	NA	NA	NA
Pseudomonas_aeruginosa_PA121617_3995	NA	NA	NA
Pseudomonas_aeruginosa_Pa1242_6732	NZ_CP022002.1:chromosome:2318071-2318394	NZ_CP022002.1:chromosome:2317713-2318078	0
Pseudomonas_aeruginosa_Pa124_6251	NA	NA	NA
Pseudomonas_aeruginosa_Pa127_6938	NA	NA	NA
Pseudomonas_aeruginosa_PA1_497	NC_022808.2:chromosome:4842694-4843017	NC_022808.2:chromosome:4843010-4843375	0
Pseudomonas_aeruginosa_PA_150577_6347	NZ_CP017306.1:chromosome:4666303-4666626	NZ_CP017306.1:chromosome:4666619-4666984	0
Pseudomonas_aeruginosa_PA_154197_5532	NA	NA	NA
Pseudomonas_aeruginosa_PA1_7599	NC_022808.1:chromosome:4799984-4800307	NC_022808.1:chromosome:4799626-4799991	0
Pseudomonas_aeruginosa_PA1R_496	NC_022806.1:chromosome:4799984-4800307	NC_022806.1:chromosome:4799626-4799991	0
Pseudomonas_aeruginosa_PA1RG_3201	NZ_CP012679.1:chromosome:4842695-4843018	NZ_CP012679.1:chromosome:4843011-4843376	0

Pseudomonas_aeruginosa_PA298_11122	NZ_CP040127.1:chromosome:4782878-4783201	NZ_CP040127.1:chromosome:4783194-4783559	0
Pseudomonas_aeruginosa_PA34_9522	NA	NA	NA
Pseudomonas_aeruginosa_Pa58_6334	NZ_CP021775.1:chromosome:5432926-5433249	NZ_CP021775.1:chromosome:5433242-5433607	0
Pseudomonas_aeruginosa_PA59_12042	NZ_CP024630.1:chromosome:4974533-4974856	NZ_CP024630.1:chromosome:4974849-4975214	0
Pseudomonas_aeruginosa_PA7_119	NA	NA	NA
Pseudomonas_aeruginosa_PA7790_5368	NZ_CP014999.1:strain:5034891-5035214	NZ_CP014999.1:strain:5035207-5035572	0
Pseudomonas_aeruginosa_PA8281_5357	NZ_CP015002.1:chromosome:5012556-5012879	NZ_CP015002.1:chromosome:5012872-5013237	0
Pseudomonas_aeruginosa_PA83_6078	NA	NA	NA
Pseudomonas_aeruginosa_Pa84_6035	NZ_CP021999.1:chromosome:4826774-4827097	NZ_CP021999.1:chromosome:4827090-4827455	0
Pseudomonas_aeruginosa_PABL012_9632	NZ_CP031659.1:chromosome:4804875-4805198	NZ_CP031659.1:chromosome:4805191-4805556	0
Pseudomonas_aeruginosa_PABL017_9633	NZ_CP031660.1:chromosome:4769939-4770262	NZ_CP031660.1:chromosome:4770255-4770620	0
Pseudomonas_aeruginosa_PABL048_9601	NA	NA	NA
Pseudomonas_aeruginosa_PACS2_84	NA	NA	NA
Pseudomonas_aeruginosa_PA_D1_3998	NZ_CP012585.1:chromosome:4923959-4924282	NZ_CP012585.1:chromosome:4924275-4924640	0
Pseudomonas_aeruginosa_PA_D16_4001	NZ_CP012581.1:chromosome:4962111-4962434	NZ_CP012581.1:chromosome:4962427-4962792	0
Pseudomonas_aeruginosa_PA_D21_4006	NZ_CP012582.1:chromosome:4919245-4919568	NZ_CP012582.1:chromosome:4919561-4919926	0
Pseudomonas_aeruginosa_PA_D22_4002	NZ_CP012583.1:chromosome:4962117-4962440	NZ_CP012583.1:chromosome:4962433-4962798	0
Pseudomonas_aeruginosa_PA_D2_3999	NZ_CP012578.1:chromosome:4923132-4923455	NZ_CP012578.1:chromosome:4923448-4923813	0
Pseudomonas_aeruginosa_PA_D25_4003	NZ_CP012584.1:chromosome:4963342-4963665	NZ_CP012584.1:chromosome:4963658-4964023	0
Pseudomonas_aeruginosa_PA_D5_4005	NZ_CP012579.1:chromosome:4962128-4962451	NZ_CP012579.1:chromosome:4962444-4962809	0
Pseudomonas_aeruginosa_PA_D9_4000	NZ_CP012580.1:chromosome:4925613-4925936	NZ_CP012580.1:chromosome:4925929-4926294	0
Pseudomonas_aeruginosa_PAER4_119_5012	NZ_CP013113.1:chromosome:4820370-4820693	NZ_CP013113.1:chromosome:4820686-4821051	0

Pseudomonas_aeruginosa_PAK_12154	NA	NA	NA
Pseudomonas_aeruginosa_PAK_6441	NA	NA	NA
Pseudomonas_aeruginosa_PAO1_107	NA	NA	NA
Pseudomonas_aeruginosa_PAO1161_10710	NA	NA	NA
Pseudomonas_aeruginosa_PAO1_Orsay_5354	NA	NA	NA
Pseudomonas_aeruginosa_PASGNDM345_6255	NZ_CP020703.1:chromosome:5178938-5179261	NZ_CP020703.1:chromosome:5179254-5179619	0
Pseudomonas_aeruginosa_PASGNDM699_7104	NZ_CP020704.1:chromosome:5270875-5271198	NZ_CP020704.1:chromosome:5271191-5271556	0
Pseudomonas_aeruginosa_PB350_6610	NZ_CP025055.1:chromosome:5004750-5005073	NZ_CP025055.1:chromosome:5005066-5005431	0
Pseudomonas_aeruginosa_PB353_6129	NA	NA	NA
Pseudomonas_aeruginosa_PB354_7065	NA	NA	NA
Pseudomonas_aeruginosa_PB367_7067	NZ_CP025056.1:chromosome:5004786-5005109	NZ_CP025056.1:chromosome:5005102-5005467	0
Pseudomonas_aeruginosa_PB368_7201	NZ_CP025050.1:chromosome:4271095-4271418	NZ_CP025050.1:chromosome:4271411-4271776	0
Pseudomonas_aeruginosa_PB369_7191	NZ_CP025049.1:chromosome:4238367-4238690	NZ_CP025049.1:chromosome:4238683-4239048	0
Pseudomonas_aeruginosa_PPF-1_6589	NA	NA	NA
Pseudomonas_aeruginosa_RD1-3_12124	NZ_CP047697.1:chromosome:2150573-2150896	NZ_CP047697.1:chromosome:2150215-2150580	0
Pseudomonas_aeruginosa_RIVM-EMC2982_6088	NZ_CP016955.1:chromosome:1999795-2000118	NZ_CP016955.1:chromosome:2000111-2000458	0
Pseudomonas_aeruginosa_RP73_192	NC_021577.1:chromosome:862284-862607	NC_021577.1:chromosome:861926-862291	0
Pseudomonas_aeruginosa_S86968_6031	NA	NA	NA
Pseudomonas_aeruginosa_S86968_7634	NA	NA	NA
Pseudomonas_aeruginosa_SCV20265_215	NA	NA	NA
Pseudomonas_aeruginosa_SCVFeb_5350	NA	NA	NA
Pseudomonas_aeruginosa_SCVJan_5538	NA	NA	NA
Pseudomonas_aeruginosa_SP2230_10393	NA	NA	NA
Pseudomonas_aeruginosa_SP4371_10392	NA	NA	NA
Pseudomonas_aeruginosa_SP4527_10694	NA	NA	NA
Pseudomonas_aeruginosa_SP4528_9945	NA	NA	NA

Pseudomonas_aeruginosa_ST773_12047	NZ_CP041945.1:chromosome:4992489-4992812	NZ_CP041945.1:chromosome:4992822-4993169	10
Pseudomonas_aeruginosa_T2101_12052	NZ_CP039990.1:chromosome:4784570-4784893	NZ_CP039990.1:chromosome:4784886-4785251	0
Pseudomonas_aeruginosa_T2436_12051	NA	NA	NA
Pseudomonas_aeruginosa_T38079_5952	NA	NA	NA
Pseudomonas_aeruginosa_T38079_7635	NA	NA	NA
Pseudomonas_aeruginosa_UCBPP-PA14_109	NA	NA	NA
Pseudomonas_aeruginosa_USDA-ARS-USMARC-41639_3640	NZ_CP013989.1:chromosome:4729169-4729492	NZ_CP013989.1:chromosome:4729485-4729850	0
Pseudomonas_aeruginosa_VA-134_3846	NA	NA	NA
Pseudomonas_aeruginosa_VRFPA04_230	NZ_CP008739.1:chromosome:1886188-1886511	NZ_CP008739.1:chromosome:1885830-1886195	0
Pseudomonas_aeruginosa_W16407_6873	NZ_CP008869.2:chromosome:4984581-4984904	NZ_CP008869.2:chromosome:4984897-4985262	0
Pseudomonas_aeruginosa_W16407_7636	NZ_CP008869.1:chromosome:1117295-1117618	NZ_CP008869.1:chromosome:1116937-1117302	0
Pseudomonas_aeruginosa_W36662_7266	NA	NA	NA
Pseudomonas_aeruginosa_W36662_7637	NA	NA	NA
Pseudomonas_aeruginosa_W45909_5913	NZ_CP008871.2:chromosome:4954531-4954854	NZ_CP008871.2:chromosome:4954847-4955212	0
Pseudomonas_aeruginosa_W45909_7638	NZ_CP008871.1:chromosome:1000388-1000711	NZ_CP008871.1:chromosome:1000030-1000395	0
Pseudomonas_aeruginosa_W60856_6659	NZ_CP008864.2:chromosome:1764382-1764705	NZ_CP008864.2:chromosome:1764024-1764389	0
Pseudomonas_aeruginosa_W60856_7639	NZ_CP008864.1:chromosome:416026-416349	NZ_CP008864.1:chromosome:415668-416033	0
Pseudomonas_aeruginosa_WCHPA075019_6412	NZ_CP028584.1:chromosome:5098897-5099220	NZ_CP028584.1:chromosome:5099213-5099578	0
Pseudomonas_aeruginosa_WCHPA075019_9521	NZ_CP028584.2:chromosome:5098897-5099220	NZ_CP028584.2:chromosome:5099213-5099578	0
Pseudomonas_aeruginosa_X78812_6522	NA	NA	NA
Pseudomonas_aeruginosa_X78812_7644	NA	NA	NA
Pseudomonas_aeruginosa_Y31_6550	NA	NA	NA
Pseudomonas_aeruginosa_Y71_9532	NZ_CP030911.1:chromosome:4990210-4990533	NZ_CP030911.1:chromosome:4990526-4990891	0

Pseudomonas_aeruginosa_Y82_7124	NZ_CP030912.1:chromosome:5889781-5890104	NZ_CP030912.1:chromosome:5890097-5890444	0
Pseudomonas_aeruginosa_Y89_7172	NZ_CP030913.1:chromosome:4927126-4927449	NZ_CP030913.1:chromosome:4927442-4927807	0
Pseudomonas_aeruginosa_YB01_12114	NZ_CP028132.1:chromosome:4598419-4598742	NZ_CP028132.1:chromosome:4598735-4599100	0
Pseudomonas_aeruginosa_YL84_2501	NZ_CP007147.1:chromosome:2522961-2523284	NZ_CP007147.1:chromosome:2522603-2522968	0

[a]The genomic distance between the *prrT-prrA* homologs, the 0 value indicates gene overlap

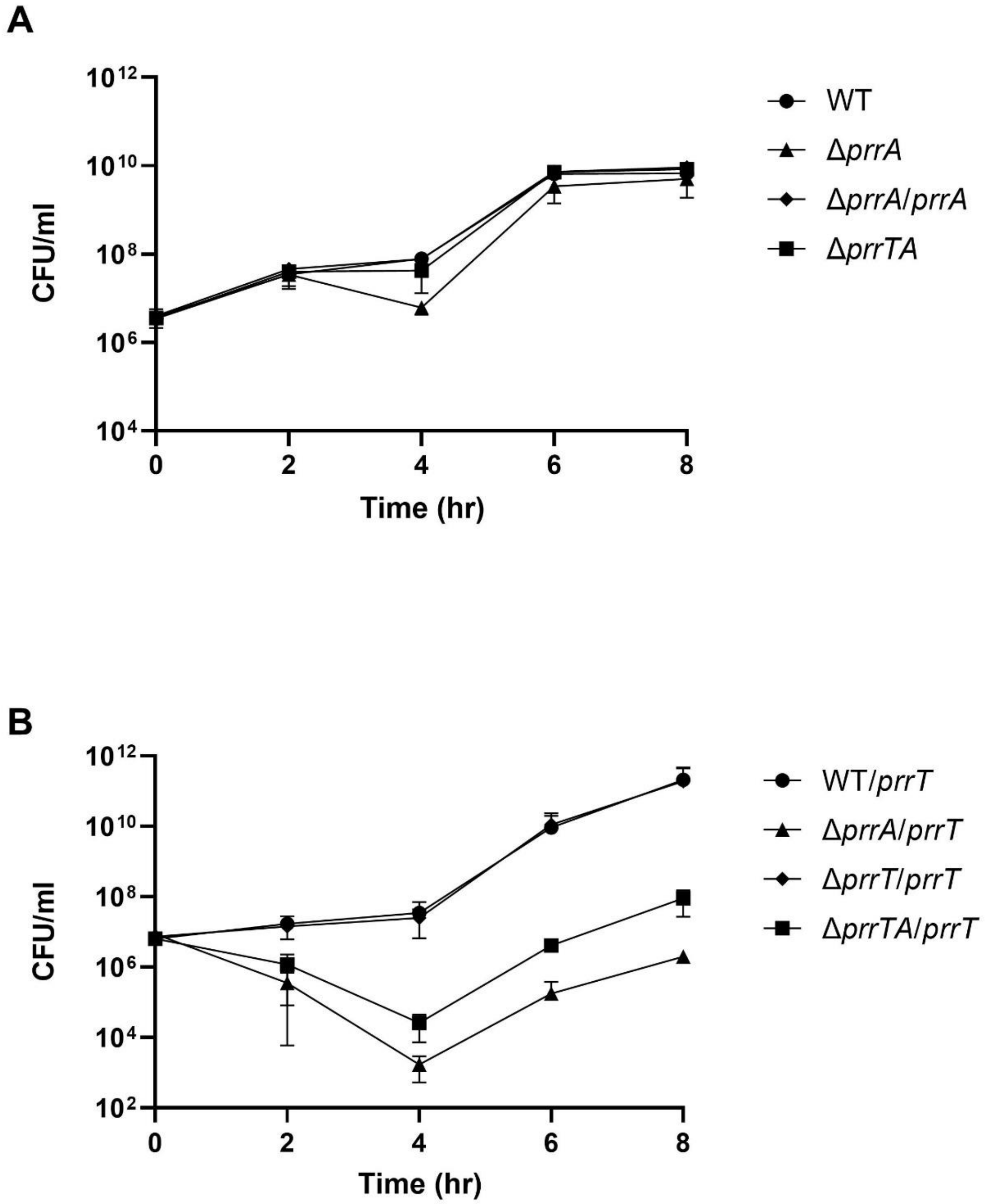
A
10^{12}
10^{10}
10^{8}
10^{6}
10^{4}
CFU/ml
0
2
4
6
8
Time (hr)
WT
Δ*prrA*
Δ*prrA*/*prrA*
Δ*prrTA*
B
10^{12}
10^{10}
10^{8}
10^{6}
10^{4}
10^{2}
CFU/ml
0
2
4
6
8
Time (hr)
WT/*prrT*
Δ*prrA*/*prrT*
Δ*prrT*/*prrT*
Δ*prrTA*/*prrT*

Figure S1

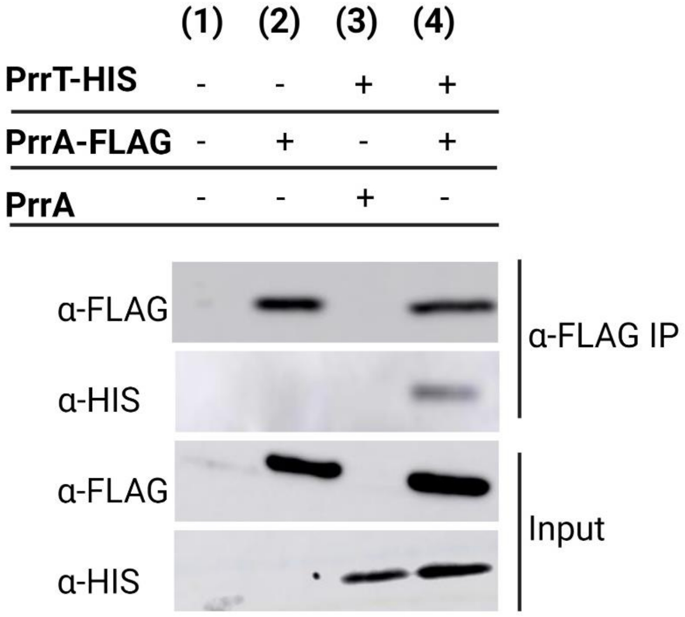

Figure S2

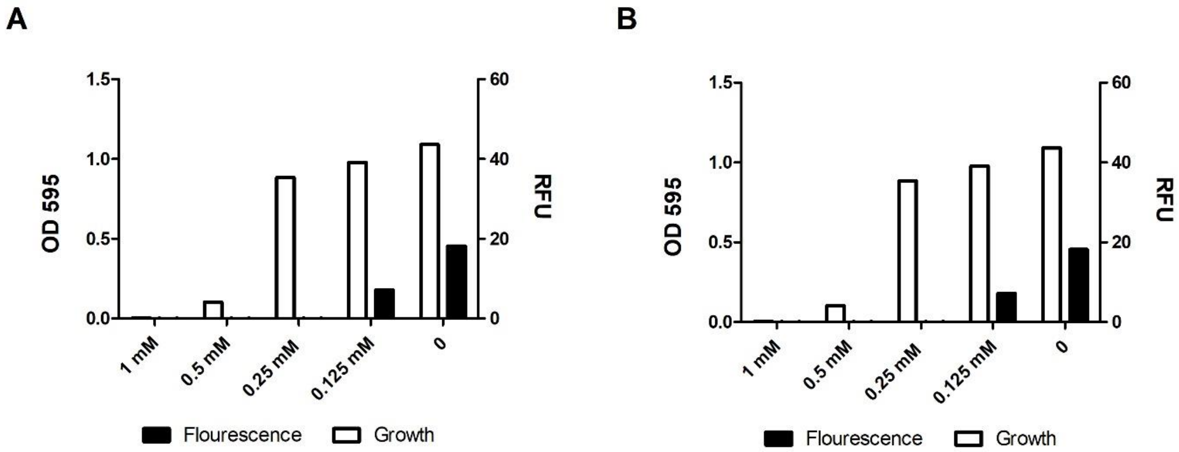

Figure S3

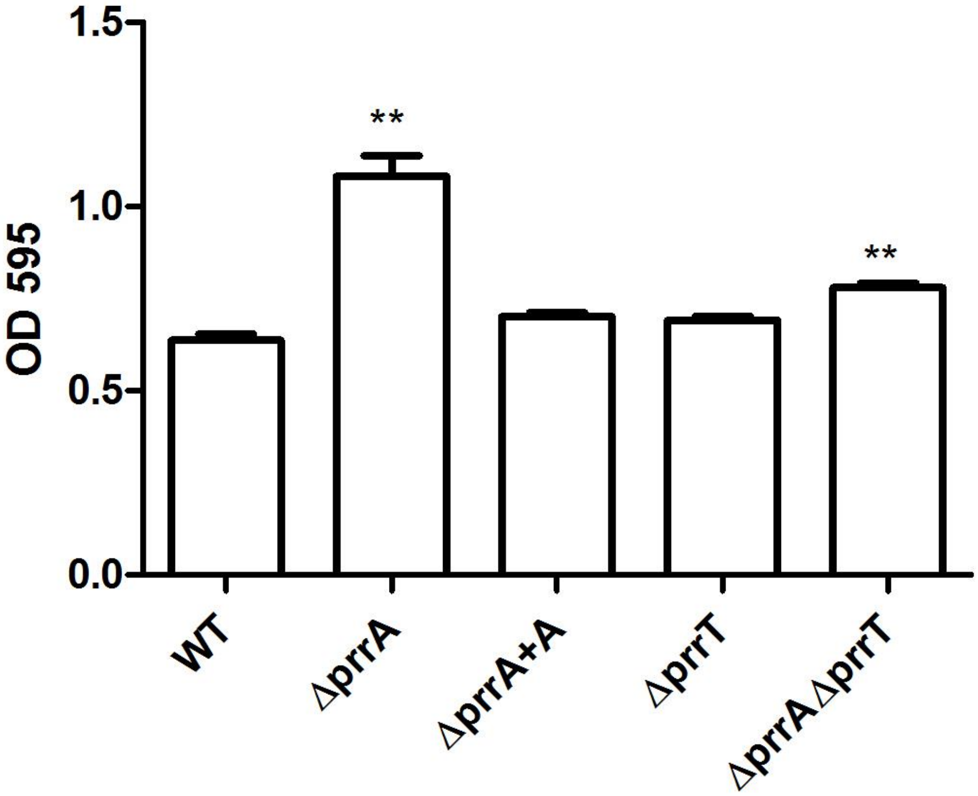

Figure S4

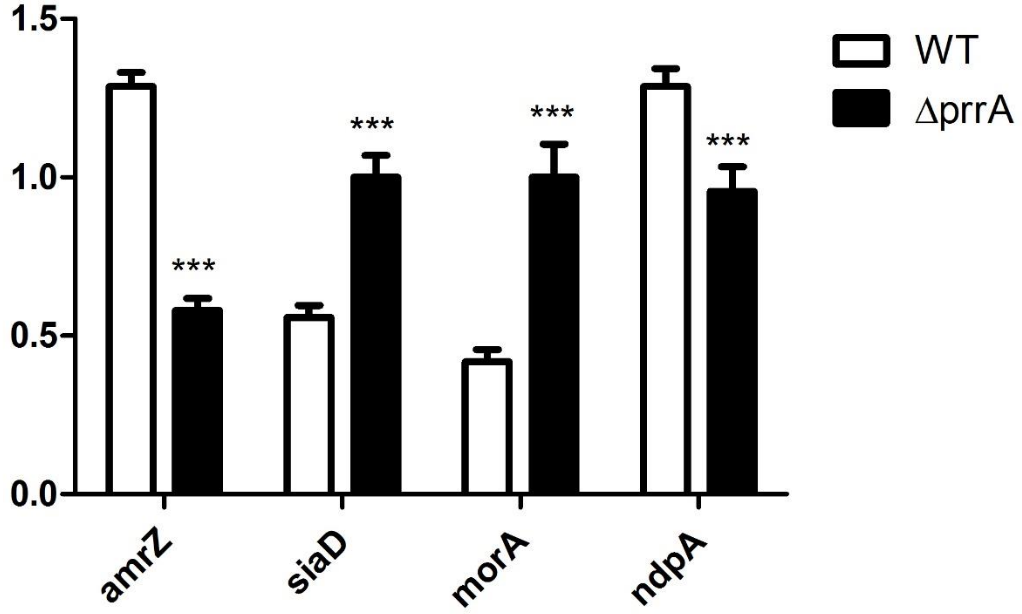

Figure S5

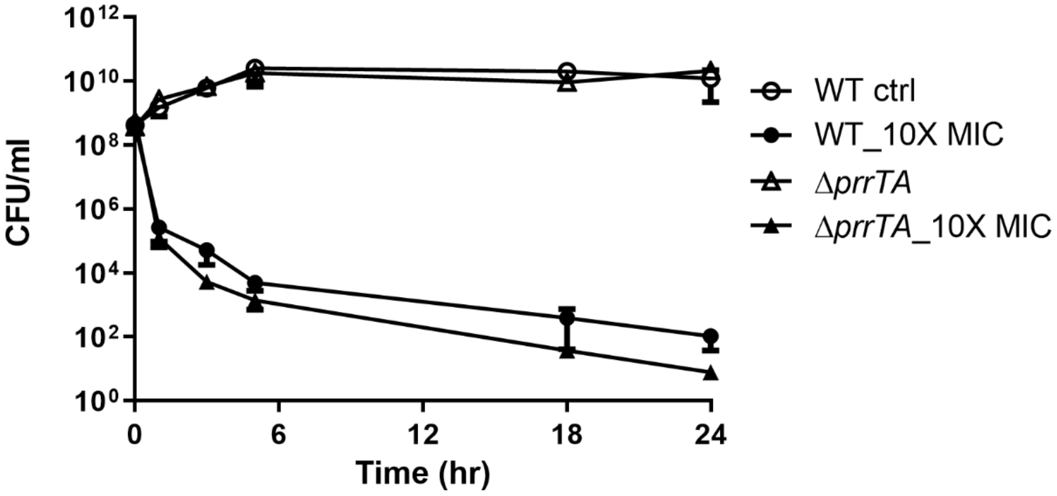

Figure S6

Supplementary references

1. Wozniak DJ, Ohman DE. 1991. *Pseudomonas aeruginosa* AlgB, a two-component response regulator of the NtrC family, is required for algD transcription. J Bacteriol 173: 1406–1413.

2. He J, Baldini RL, Déziel E, Saucier M, Zhang Q, Liberati NT, Lee D, Urbach J, Goodman HM, Rahme LG. 2004. The broad host range pathogen *Pseudomonas aeruginosa* strain PA14 carries two pathogenicity islands harboring plant and animal virulence genes. Proc Natl Acad Sci U S A 101: 2530–2535..

3. Simon R, Priefer U, Puhler A. 1983. A broad host range mobilization system for in vivo genetic engineering: transposon mutagenesis in gram negative bacteria. Nat Biotechnol 784–791.

4. Karimova G, Pidoux J, Ullmann A, Ladant D. 1998. A bacterial two-hybrid system based on a reconstituted signal transduction pathway. Proc Natl Acad Sci U S A 95:5752-5756.

5. Schweizer HP. 1991. *Escherichia-Pseudomonas* shuttle vectors derived from pUC18/19. Gene 97:109–112.

6. Laura R. Hmelo BR et al K. 2016. Precision-engineering the *Pseudomonas aeruginosa* genome with two-step allelic exchange. Nat Rev Drug Discov 5:1–8.

7. Becher A, Schweizer HP. 2000. Integration-proficient *Pseudomonas aeruginosa* vectors for isolation of single-copy chromosomal *lacZ* and *lux* gene fusions. Biotechniques 29:948-50.

8. Choi KH, Schweizer HP. 2006. mini-Tn7 insertion in bacteria with single *attTn7* sites: Example *Pseudomonas aeruginosa*. Nat Protoc 1**:**153–161.

9. Newman JR, Fuqua C. 1999. Broad-host-range expression vectors that carry the L-arabinose-inducible *Escherichia coli* araBAD promoter and the araC regulator. Gene 227:197–203.
